# Correction: Data Simulation in Machine Olfaction with the R Package *Chemosensors*

**DOI:** 10.1371/journal.pone.0097796

**Published:** 2014-05-09

**Authors:** The PLOS ONE Staff

## Notice of Republication

This article was republished on April 22, 2014, to correct errors in Figures 3, 4, and 5 that were introduced during the production process. Please download this article again to view the correct figures.

PLOS style does not allow for the code in the PDF version of this article to be displayed as the authors intended. Please see the author-provided PDF attached to this notice to view the correctly formatted code.

The publisher apologizes for the figure errors and the code formatting in the PLOS PDF.

The originally published, uncorrected article and the republished article with corrected figures are also provided here for reference.

## Supporting Information

File S1 Originally published, uncorrected article.
(PDF)

File S2 Republished article with corrected figures.
(PDF)

File S3 Author-provided PDF.
(PDF)
