# Supplementary material for: Data Simulation in Machine Olfaction with the R Package Chemosensors
Source: PLoS One. 2014 Feb 26;9(2):e88839. doi: 10.1371/journal.pone.0088839 (PMC3935855; doi:10.1371/journal.pone.0088839)
Supplement: File S1 — Ten scenarios for machine olfaction in the framework of the NEUROChem project [3] . (PDF) [file pone.0088839.s001.pdf]

File S1: Ten scenarios for machine olfaction in the framework of the NEUROChem project

Andrey Ziyatdinov, Alexandre Perera-Lluna

21/01/2014

- About
- Scenarios
  - Classification
  - Quantification
  - Segmentation
  - Habituation
  - Event Detection
  - Novelty Detection
  - Drift Compensation I
  - Drift Compensation II
  - Sensor Replacement I
  - Sensor Replacement II
- Session Information

About

This documents introduces a list of ten scenarios for machine olfaction, which were initialy thought in the framework of the NEUROChem project. Each scenario is described in terms of training and validation sets and scenario difficulty.

The R code to create an object of `Scenario` class is given for all ten scenarios, except two scenarios Habituation and Event Detection. The point is that `Scenario` class is not suitable for these two scenarios, because the given class is thought to be constructed only by means of gas pulses, while the two scenarios require another time profile.

First of all, the user needs to load the package.

```
library(chemosensors)
```

An object of `Scenario` class is initialized with `Scenario` function, that has the following parameters:

- `tunit`: the length of the pulse.
- `concUnits`: the concentration units.
- `randomize`: whether the gas classes need to be randomized in order.
- `T` and `nT`: gas classes for the training set and the number of repetitions for each class.
- `V` and `nV`: gas classes for the training set and the number of repetitions for each class.

In addition to the initialization code, the results of `print` and `plot` methods for objects of `Scenario` class are shown.

Scenarios

Classification

John has three vessels with three odours A, B, C. The system is trained with all three compounds separately. John approaches the vessel B to the system. The machine identifies correctly odour B. The difficulty is the similarity between the odours to be identified.

```
sc.class <- Scenario(name = "Classification",
  tunit = 60, concUnits = "norm", randomize = TRUE,
  T = c("A", "B", "C"), nT = 30, V = "B", nV = 30)
```

```
sc.class
```

```
##  Scenario `Classification` of 120 samples, tunit 60, randomize TRUE
##  - gases A, B, C
##  - Training Set: A (30), B (30), C (30)
##  - Validation Set: B (30)
```

```
plot(sc.class)
```

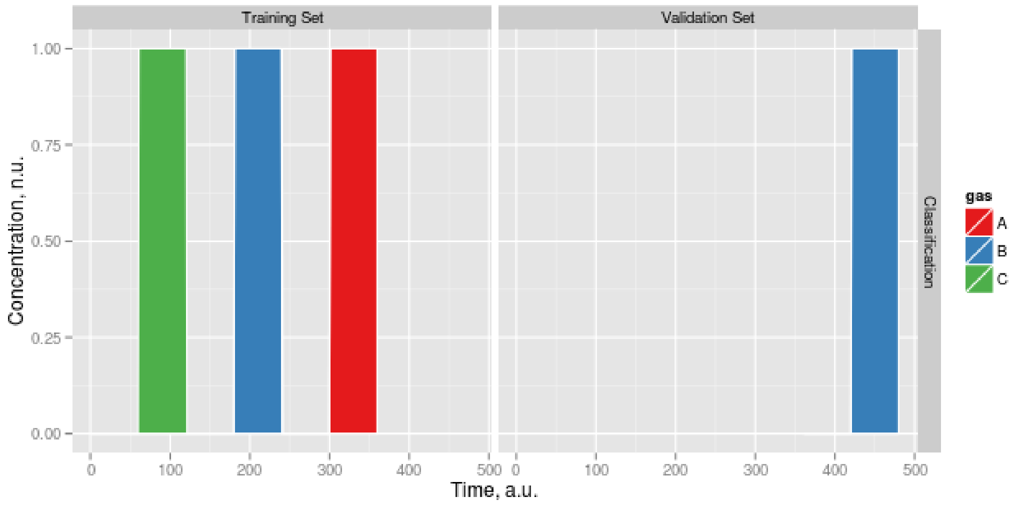

Quantification

John has five vessels with 100%, 50%, 10% and 1% dilution of A. The system is trained with 100%, 10% and 1% dilution of A. John approaches the 50% A dilution vessel to the system. The machine correctly marks the level of A. The difficulty is the number of different concentration examples available for training.

```
sc.quant <- Scenario(name = "Quantification",
  tunit = 60, concUnits = "norm", randomize = TRUE,
  T = c("A 0.01", "A 0.1", "A"), nT = 30, V = "A 0.5", nV = 30)

sc.quant
```

```
##  Scenario `Quantification` of 120 samples, tunit 60, randomize TRUE
##  - gases A, B, C
##  - Training Set: A (30), A 0.01 (30), A 0.1 (30)
##  - Validation Set: A 0.5 (30)
```

```
plot(sc.quant)
```

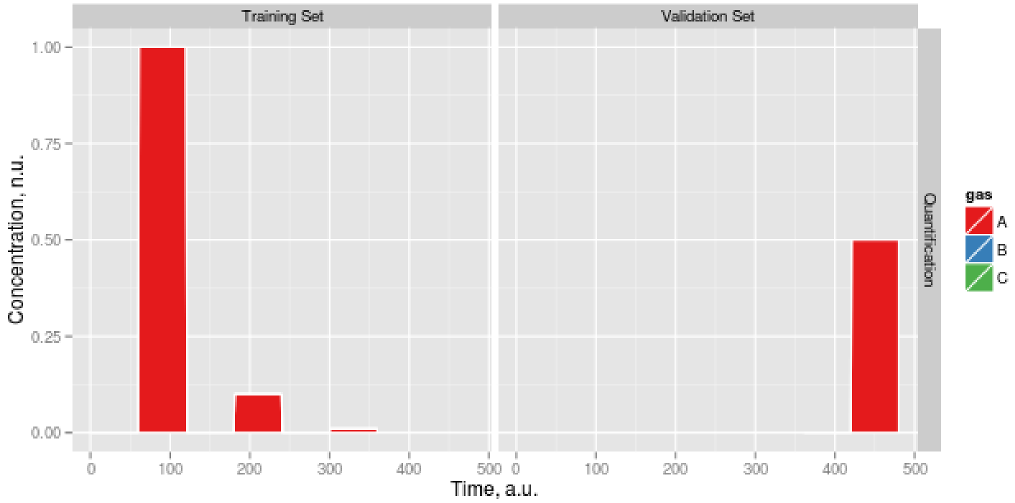

Segmentation

John has three vessels with three odours A, B and C. The system is trained with all three compounds separately. John approaches vessel B to the system. The machine identifies correctly odour B. John approaches A+B to the system. The machine identifies A and identifies B sequentially. The difficulty is the similarity between the odours to be segmented.

```
sc.seg <- Scenario(name = "Segmentation",
  tunit = 60, concUnits = "norm", randomize = TRUE,
  T = c("A", "B", "C"), nT = 30, V = c("B", "A 0.5, B 0.5"), nV = 30)

sc.seg
```

```
##  Scenario `Segmentation` of 150 samples, tunit 60, randomize TRUE
##  - gases A, B, C
##  - Training Set: A (30), B (30), C (30)
##  - Validation Set: A 0.5, B 0.5 (30), B (30)
```

```
plot(sc.seg)
```

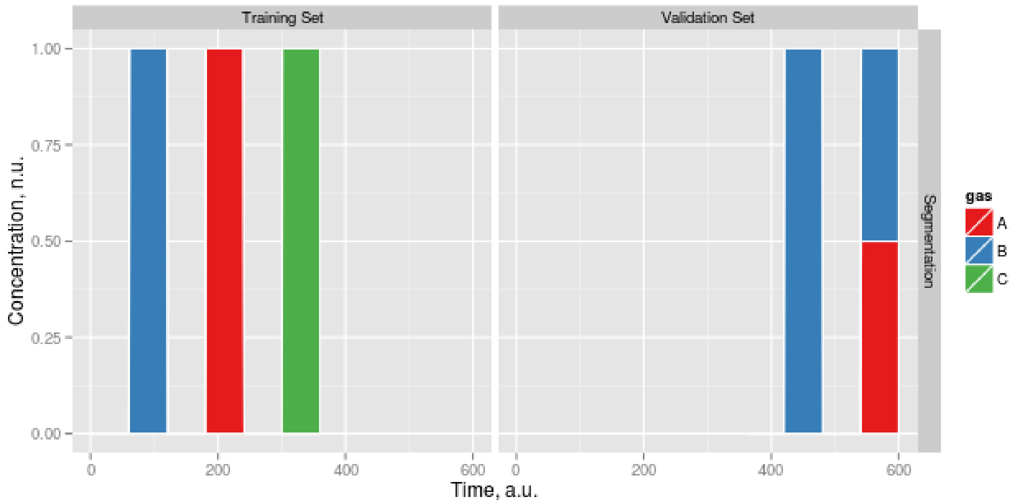

Habituation

John has three vessels with three odours A, B, C. The system is trained with all three compounds separately. John approaches vessel A to the system. The machine identifies vessel A. After a certain time the machine marks that no odour is present despite the vessel is still exposed to the system. The difficulty is the concentration of odour A, as the higher the concentration the more difficult is to adapt to the odour.

```
sc.hab <- Scenario(name = "Habituation",
  tunit = 60, concUnits = "norm", randomize = TRUE,
  T = c("A", "B", "C"), nT = 30, V = "A", nV = 30)

sc.hab
```

```
##  Scenario `Habituation` of 120 samples, tunit 60, randomize TRUE
##  - gases A, B, C
##  - Training Set: A (30), B (30), C (30)
##  - Validation Set: A (30)
```

```
plot(sc.hab)
```

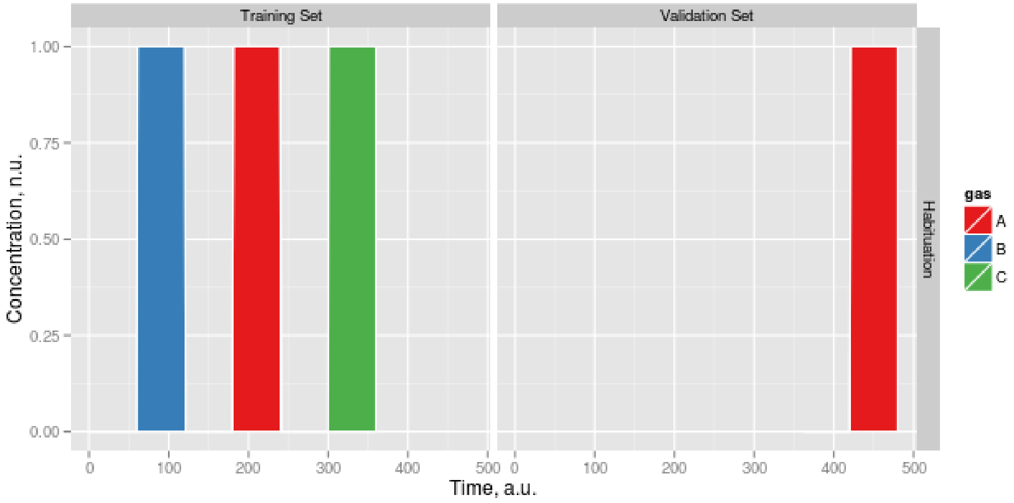

Event Detection

The system is not trained with any compound. No substance is exposed to the machine. The machine marks that no odour is present. John approaches the vessel B to the system. The machine marks that one odour is present. John approaches the vessel A, in addition to already present B vessel to the system. The machine marks that two odours are present. The difficulty is the magnitude of the second odour B added to the first odour A. The first odour A will be fully delivered on a 100%.

Novelty Detection

John has two vessels with odours A and B respectively. The system is trained only with odour A. No substance is exposed to the machine. The machine marks that no odour is present. John approaches the vessel A to the system. The machine marks that odour A is present. John approaches the vessel B, in addition to already present A vessel to the system. The machine marks that a new odour is present. The difficulty is the concentration of odour B, as the lower the concentration of odour B the more difficult the detection of the novel odour become.

Drift Compensation I

John has three vessels with three odours A, B and C. The system is trained with all three compounds separately. John approaches the vessel B to the system. The machine identifies correctly odour B. A drift process is occurring in the sensor array. John approaches the vessel B to the machine. The machine identifies correctly odour B. The difficulty is the distance in time between the validation set and the training set.

```
sc.drift1 <- Scenario(name = "Drift Compensation I",
  tunit = 60, concUnits = "norm", randomize = TRUE,
```

```
  T = c("A", "B", "C"), nT = 30, V = "B", nV = 30)

sc.drift1
```

```
##  Scenario `Drift Compensation I` of 120 samples, tunit 60, randomize TRUE
##  - gases A, B, C
##  - Training Set: A (30), B (30), C (30)
##  - Validation Set: B (30)
```

```
plot(sc.drift1)
```

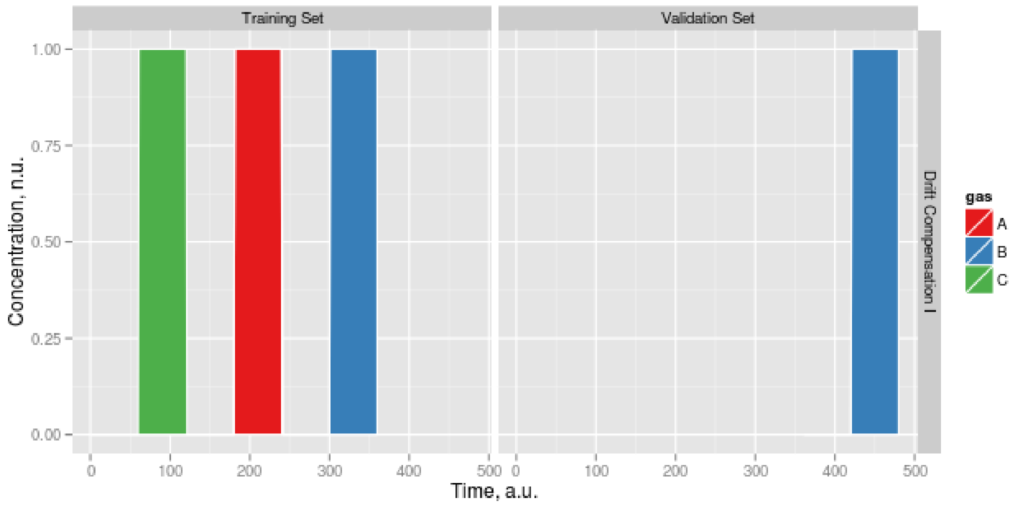

Drift Compensation II

John has five vessels with 100%, 50%, 10% and 1% dilution of A. The system is trained with 100%, 10% and 1% dilution of A. A drift process is induced into the sensor array. John approaches the 50% A dilution vessel to the system. The machine correctly marks the level of A. The difficulty is the distance in time between the validation set and the training set.

```
sc.drift2 <- Scenario(name = "Drift Compensation II",
  tunit = 60, concUnits = "norm", randomize = TRUE,
  T = c("A 0.01", "A 0.1", "A"), nT = 30, V = "A 0.5", nV = 30)

sc.drift2
```

```
##  Scenario `Drift Compensation II` of 120 samples, tunit 60, randomize TRUE
##  - gases A, B, C
##  - Training Set: A (30), A 0.01 (30), A 0.1 (30)
##  - Validation Set: A 0.5 (30)
```

```
plot(sc.drift2)
```

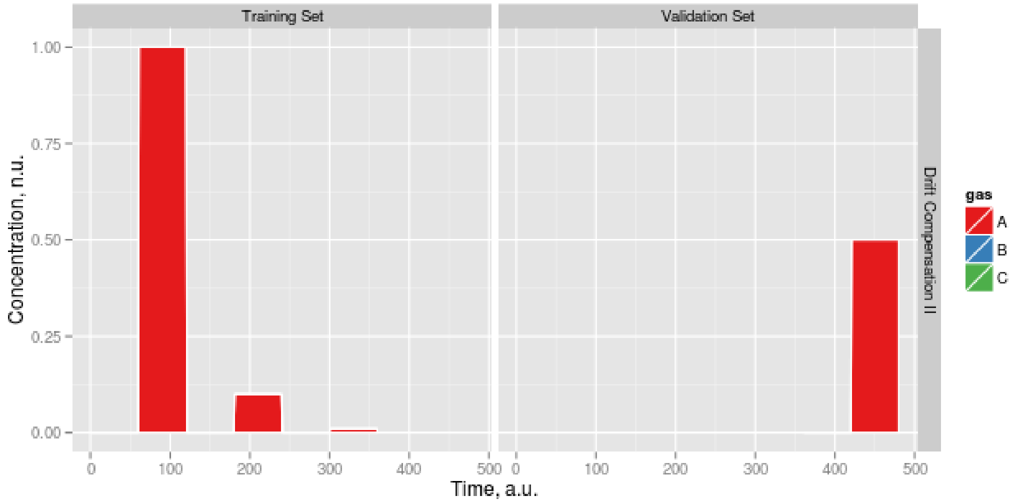

Sensor Replacement I

John has three vessels with three odours A, B and C. The system is trained with all three compounds separately. John approaches vessel B to the system. The machine identifies correctly odour B. A certain proportion of specific sensors in the array are (virtually) damaged, so John replaces them with new sensors of the same type. John approaches vessel B to the system. The machine identifies correctly odour B without new training. The dificulty is the proprtion of sensors to be replaced.

```
sc.replace1 <- Scenario(name = "Sensor Replacement I",
  tunit = 60, concUnits = "norm", randomize = TRUE,
  T = c("A", "B", "C"), nT = 30, V = "B", nV = 30)

sc.replace1
```

```
##  Scenario `Sensor Replacement I` of 120 samples, tunit 60, randomize TRUE
##  - gases A, B, C
##  - Training Set: A (30), B (30), C (30)
##  - Validation Set: B (30)
```

```
plot(sc.replace1)
```

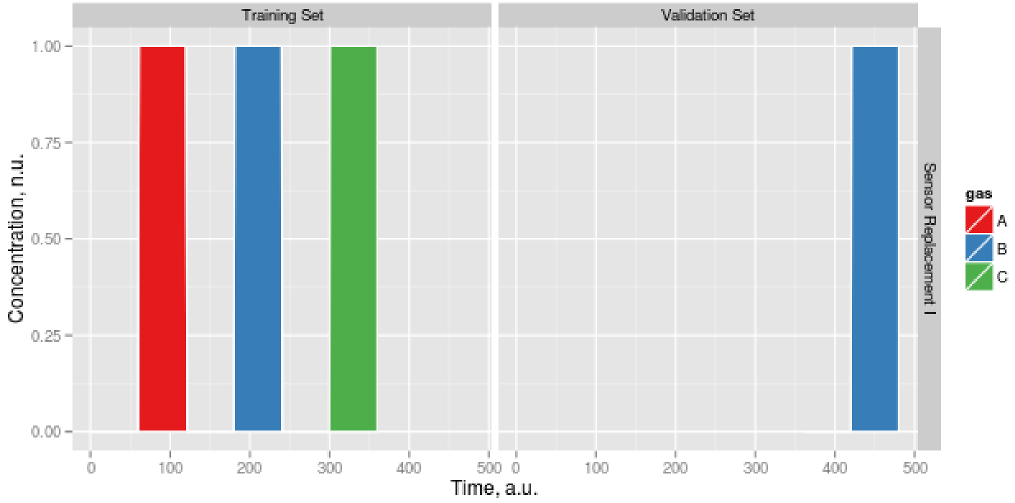

Sensor Replacement II

John has three vessels with three odours A, B and C. The system is trained with all three compounds separately. John approaches vessel B to the system. The machine identifies correctly odour B. A certain proportion of sensors in the array are (virtually) damaged, so John replaces them with new sensors. John approaches vessel B to the system. The machine identifies correctly odour B without new training. The dificulty is the proprtion of sensors to be replaced.

```
sc.replace2 <- Scenario(name = "Sensor Replacement II",
  tunit = 60, concUnits = "norm", randomize = TRUE,
  T = c("A", "B", "C"), nT = 30, V = "B", nV = 30)

sc.replace2
```

```
##  Scenario `Sensor Replacement II` of 120 samples, tunit 60, randomize TRUE
##  - gases A, B, C
##  - Training Set: A (30), B (30), C (30)
##  - Validation Set: B (30)
```

```
plot(sc.replace2)
```

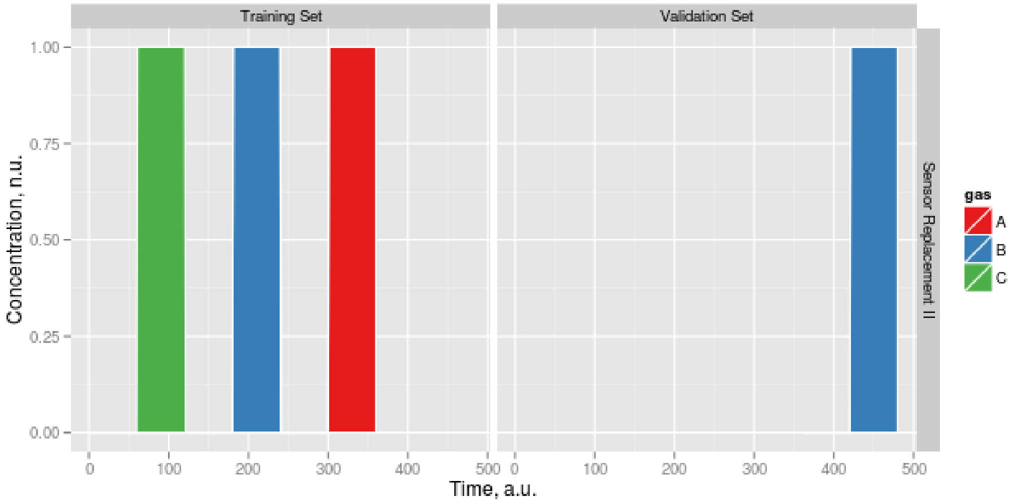

Session Information

```
sessionInfo()
```

```
## R version 3.0.1 (2013-05-16)
## Platform: x86_64-pc-linux-gnu (64-bit)
##
## locale:
##  [1] LC_CTYPE=en_US.UTF-8       LC_NUMERIC=C
##  [3] LC_TIME=en_US.UTF-8        LC_COLLATE=en_US.UTF-8
##  [5] LC_MONETARY=en_US.UTF-8    LC_MESSAGES=en_US.UTF-8
##  [7] LC_PAPER=C                 LC_NAME=C
##  [9] LC_ADDRESS=C               LC_TELEPHONE=C
## [11] LC_MEASUREMENT=en_US.UTF-8 LC_IDENTIFICATION=C
##
## attached base packages:
## [1] grid      stats     graphics  grDevices utils     datasets  methods
## [8] base
##
```

```
## other attached packages:
##  [1] chemosensors_0.7.8 pls_2.3-0          gridExtra_0.9.1   
##  [4] ggplot2_0.9.3.1.99 reshape2_1.2.2     plyr_1.8          
##  [7] ascii_2.1          xtable_1.7-1       knitr_1.3.3       
## [10] devtools_1.4.1.99 
## 
## loaded via a namespace (and not attached):
##  [1] codetools_0.2-8    colorspace_1.2-2   dichromat_2.0-0   
##  [4] digest_0.6.3       evaluate_0.4.4     formatR_0.8       
##  [7] gtable_0.1.2       httr_0.2           labeling_0.2      
## [10] LearnBayes_2.12    MASS_7.3-27        memoise_0.1       
## [13] munsell_0.4        parallel_3.0.1     proto_0.3-10      
## [16] quadprog_1.5-5     RColorBrewer_1.0-5 RCurl_1.95-4.1    
## [19] scales_0.2.3       stringr_0.6.2      tools_3.0.1       
## [22] whisker_0.3-2
```
