# Supplementary material for: Data Simulation in Machine Olfaction with the R Package Chemosensors
Source: PLoS One. 2014 Feb 26;9(2):e88839. doi: 10.1371/journal.pone.0088839 (PMC3935855; doi:10.1371/journal.pone.0088839)
Supplement: Appendix S1 — Parameterization of Simulation Models. (PDF) [file pone.0088839.s002.pdf]

Appendix S1: Parameterization of Simulation Models

Andrey Ziyatdinov[1,2], Alexandre Perera-Lluna[1,2]
1 Department of ESAII, Universitat Politènica de Catalunya, Pau Gargallo 5, Barcelona, Spain
2 Centro de Investigación Biomèdica en Red en Bioingeniería, Biomateriales y Nanomedicina (CIBER-BBN), Barcelona, Spain

The virtual sensors available in *chemosensors* package are derived from the seventeen UNIMAN sensors based on the model parameters computed for the UNIMAN data set in [1]. In this section, we briefly review the simulation models and their parameters, in order to demonstrate the mechanism of creating virtual sensors.

The sorption model defined in Equation 1 establishes a relation between the environmental concentrations of analytes C_{0i} and the concentrations of analytes C_i when adsorbed by the sensor device. This relationship underlines the Langmuir isotherm for a multi-component gas mixture with two parameters for each analyte i, sorption capacity Q_i and sorption affinity K_i [2].

$$C_i = \frac{Q_i \; K_i \; C_{0i}}{1 + \sum_{j=1}^{3} K_j \; C_{0j}}, \; i \; = 1, \; 2, \; 3 \tag{1}$$

The parameters of the sorption model can be used to control such characteristics of virtual sensors as non-linearity and affinity to analytes in a mixture.

- The *Non-linearity* of a sensor depends on a relation between the numerator and the denominator in the equation. Smaller values of the affinity coefficients K_i make the denominator closer to one, resulting in linear behaviour of the sensor. On the contrary, greater values of K_i lead to saturation mode, where the magnitude of the output concentrations does not depend on the input concentrations.
- The *Affinity* property of a sensor to analyte i in a mixture is controlled by parameter K_i, and is to be estimated by comparison with the affinities for the other analytes.

A static calibration model was defined in Equations (2) and (3) in [1] and simulated the steady-state signal x_{ss} of a sensor in response to the concentrations C_i derived from the sorption model. The calibration model explicitly assumed that the response to a mixture of analytes is the sum of the individual responses to analytes. The main parameters of the model were the sensitivity coefficients $\beta_{i,k}$ to analyte i on the concentration interval k. The calibration model defines such characteristics of a virtual sensor as its sensitivity, selectivity and diversity.

- The *Sensitivity* coefficients β_i give a quantitative estimate of how sensitive a sensor is in response to the analyte i on the given concentration interval k.
- The *Selectivity* of a sensor across two analytes i and j can be evaluated by comparing the sensitivity coefficients β_i and β_j along the analytes.
- The *Diversity* property of a group of sensors is related to the redundancy of the sensor sensitivity coefficient β, and is to be estimated by some multi-variate method.

A dynamic calibration model was defined in Equation (5) in [1] and described the dynamic part of the calibration model. The model derived the temporal signal $x(t)$ from the steady state value x_{ss}. The model had two time constants per analyte as parameters, $\tau_{1,i}$ and $\tau_{2,i}$ for the analyte i. The transient model was rather simple, and we suggest relying on the steady state feature of the signal x_{ss}, rather than on transient features which could be extracted from the signal $x(t)$.

In summary, the sorption and calibration models simulate the seventeen UNIMAN sensors by a set of parameters K_i, $\beta_{i,k}$, $\tau_{1,i}$ and $\tau_{2,i}$ for each sensor. When one defines an array of virtual sensors in the *chemosensors* package, the UNIMAN sensors are replicated by varying the parameters of the simulation models. Parameters $\beta_{i,k}$, $\tau_{1,i}$ and $\tau_{2,i}$ are generated from univariate uniform distributions with control for non-negative values and the level of spread. The parameters K_i are estimated from the seventeen UNIMAN profiles, this allows preservation of the intrinsic number of sensor types given in the reference UNIMAN data set. Hence, one can imagine a virtual sensor as a *replica* of one of the seventeen UNIMAN sensors with similar characteristics on their sensitivity and selectivity profiles, the dynamic ranges for the three analytes and their signal-to-noise performance. The diversity of sensors come from two sources: the relationship between sensors found the reference UNIMAN data set and the distribution of $\beta_{i,k}$ coefficients.

The second group of simulation models defined three types of noise to be injected into the sensor signals. These types were characterised as additive, multiplicative and common noise, corresponding respectively to the concentration, sensor and drift noise models. Data in all three noise models were generated by means of a multi-variate normal distribution of independent variables with diagonal covariance Σ-matrices and zero mean, as shown in Equations (6), (7) and (8) in [1].

The concentration noise model defined the noise term ΔC_0 to be added to the matrix of analyte concentrations C_0. The data in the columns of the matrix ΔC_0 corresponded to the analytes A, B and C, and were derived from the normal distribution with zero mean and diagonal covariance matrix Σ_c. The diagonal form of the covariance matrix underlined the fact that the analytes do not interact with each other.

The sensor noise model generated noise in the sensitivity coefficients $\beta_{i,k}$ from the calibration model. A one-dimensional random walk based on the normal distribution with zero-mean and a single parameter, the standard deviation $\sigma_{i,k}$, was used for analyte i on the concentration interval k.

The drift noise model defined the drift noise ΔX_P to be injected into the matrix of sensor array data X in a multi-variate manner which consisted of several steps. Firstly, a drift-related subspace P was computed by means of Common Principal Component Analysis (CPCA) [3]. Secondly, the noise ΔX_P within this subspace P was generated via a random walk. A multi-dimensional random walk based on a multi-variate normal distribution with zero mean and diagonal covariance matrix Σ_d was used. Thirdly, the generated noise ΔX_P was induced by means of the inverse component correction method [1].

The magnitude and the structure of the noise in the noise models are mainly controlled by the three standard deviation parameters, along with some other parameters.

References

1. Ziyatdinov A, Fernández Diaz E, Chaudry A, Marco S, Persaud K, et al. (2013) A software tool for large-scale synthetic experiments based on polymeric sensor arrays. Sensors and Actuators B: Chemical 177: 596–604.

2. Bai R, Yang RT (2003) Improved Multisite Langmuir Model for Mixture Adsorption Using Multiregion Adsorption Theory. Langmuir : 2776–2781.

3. Ziyatdinov A, Marco S, Chaudry A, Persaud K, Caminal P, et al. (2010) Drift compensation of gas sensor array data by common principal component analysis. Sensors and Actuators B: Chemical 146: 460–465.
