# Supplementary material for: Data Simulation in Machine Olfaction with the R Package Chemosensors
Source: PLoS One. 2014 Feb 26;9(2):e88839. doi: 10.1371/journal.pone.0088839 (PMC3935855; doi:10.1371/journal.pone.0088839)
Supplement: Appendix S2 — Quantitative Comparison with Chemical Sensor Data. (PDF) [file pone.0088839.s003.pdf]

Appendix S2: Quantitative Comparison with Chemical Sensor Data

Andrey Ziyatdinov[1,2], Alexandre Perera-Lluna[1,2]
1 Department of ESAII, Universitat Politènica de Catalunya, Pau Gargallo 5, Barcelona, Spain
2 Centro de Investigación Biomèdica en Red en Bioingeniería, Biomateriales y Nanomedicina (CIBER-BBN), Barcelona, Spain

We show the validity of the computational framework for the operation of a virtual sensor array by performing a quantitative comparison between the predictions of the simulation models and reference chemical sensor data. The reference data used in this study is the UNIMAN data set that contains records from the array of 17 conducting polymer sensors in response to 8 gas classes (A 0.01, A 0.02, A 0.05, B 0.01, B 0.02, B 0.05, C 0.1 and C 1.0). The simulated data set is generated from a virtual array of 17 sensors with the noise parameters `csd`, `ssd` and `dsd` set to 0.8 in response to the same gas classes. One design goal of the software tool is the inclusion of sensor specificities in the models (such as sensor drift). Therefore, the comparison between the two data sets has been conducted from two perspectives: first, the validation of the physico-chemical models emulating the responses of the 17 UNIMAN sensors, and, second, the evaluation of the deviations from the sensor responses due to the three types of noises included in the software (concentration noise, sensor noise and drift noise).

The physico-chemical model of a single conducting polymer sensor was implemented in the sorption model under the non-linear relation of the Langmuir isotherm. The so-called short-term UNIMAN data set was used to estimate the two model parameters per analyte i and per sensor, sorption capacity Q_i and sorption affinity K_i, by means of fitting linear regression models (please check [1] for further details). The goodness of the fit of the models was evaluated by means of R^2 statistics. For analyte C, these statistics do not fall below than 0.973, whereas analytes A and B show a slightly worse performance, but always above 0.779.

To show that the three noise models are able to reproduce variance observed in the long term responses of the UNIMAN sensors, we replicated the first 1000 samples of the UNIMAN data set by means of an array of 17 virtual sensors. The qualitative comparison between the two data sets can be performed by means of principal component analysis, where one can observe that the simulated data matches the variance structure of the real data in terms of the class-dependency and noise-related data features. An example of this analysis was previously presented in [1], Section 3.1, Figure 5. For a quantitative analysis, we computed mean and standard deviation statistics for each combination of sensor and gas class. Table 1 reports these statistics for all 17 sensors and for A 0.05 gas class, being the A analyte the one showing the most dispersion. We report the relative difference defined as the absolute difference between UNIMAN and simulated values divided by the UNIMAN value. By collecting the statistics on the relative errors for all combinations of sensor and gas class (136 samples), we show that the relative differences in the means are always below 14.5% (in absolute values) and have 25%, 50% and 75% quantiles equal to -0.0340, -0.0125 and 0.0036, respectively. Similarly, the relative differences in the standard deviations are always below 48.0% (in absolute values) and have 25%, 50% and 75% quantiles equal to -0.2697, 0.0001 and 0.2034, respectively. It is worth to note that the multivariate and multi-component model of drift noise is the dominant component for the long term simulation, as it is for the actual chemical sensor behavior in the UNIMAN data set.

References

1. Ziyatdinov A, Fernández Diaz E, Chaudry A, Marco S, Persaud K, et al. (2013) A software tool for large-scale synthetic experiments based on polymeric sensor arrays. Sensors and Actuators B: Chemical 177: 596–604.

Table 1. Comparison between chemical sensor and simulated data sets (gas class A 0.05).

Sensor	Mean (UNIMAN)	Mean (Simulated)	Diff. in Mean	SD (UNIMAN)	SD (Simulated)	Diff. in SD
1	9.41	10.38	-0.10	0.59	0.53	0.10
2	9.04	8.83	0.02	0.52	0.35	0.33
3	8.92	9.36	-0.05	0.58	0.58	-0.01
4	6.25	6.68	-0.07	0.24	0.21	0.12
5	8.57	9.35	-0.09	0.59	0.48	0.18
6	7.61	8.39	-0.10	0.44	0.35	0.20
7	4.65	4.84	-0.04	0.17	0.21	-0.21
8	5.61	6.14	-0.09	0.27	0.34	-0.25
9	4.34	4.54	-0.05	0.25	0.23	0.09
10	4.73	4.94	-0.04	0.24	0.27	-0.11
11	11.27	12.42	-0.10	0.65	0.42	0.36
12	11.55	12.01	-0.04	0.54	0.43	0.19
13	8.04	8.62	-0.07	0.26	0.30	-0.15
14	7.02	7.77	-0.11	0.30	0.29	0.04
15	8.98	10.22	-0.14	0.36	0.45	-0.23
16	9.89	10.59	-0.07	0.43	0.43	-0.01
17	8.99	9.38	-0.04	0.33	0.52	-0.48

The chemical sensor UNIMAN data set (1000 samples, 17 sensors, 3 analytes and 8 gas classes) is compared to its replica simulated with the *chemosensors* package. The comparison is performed by means of mean and standard deviation statistics computed for each combination of sensor and gas class. This table shows the statistics for A 0.05 gas class as this is the combination that shows the most dispersion and higher discrepancy. The relative differences are computed as the absolute difference between UNIMAN and simulated values divided by the UNIMAN value. The statistics on the relative errors collected for all combinations of sensor and gas class (17*8 = 136 samples) show that (1) the relative differences in the means are below 14.5% (in absolute values) and have 25%, 50% and 75% quantiles equal to -0.0340, -0.0125 and 0.0036, respectively; and (2) the relative differences in standard deviation are below 48.0% (in absolute values) and have 25%, 50% and 75% quantiles equal to -0.2697, 0.0001 and 0.2034, respectively. The negative sign of the relative difference in the means for almost all sensors and A 0.05 gas class indicates the direction of drift effect which tend to increase the value of the sensor responses.
