# Supplementary material for: Correction: Data Simulation in Machine Olfaction with the R Package Chemosensors
Source: PLoS One. 2014 May 9;9(5):e97796. doi: 10.1371/journal.pone.0097796 (PMC4016323; doi:10.1371/journal.pone.0097796)
Supplement: File S2 — Republished article with corrected figures. (PDF) [file pone.0097796.s002.pdf]

PLOS ONE

Data Simulation in Machine Olfaction with the R Package *Chemosensors*

Andrey Ziyatdinov[1,2]*, Alexandre Perera-Lluna[1,2]

1 Department of ESAII, Universitat Politènica de Catalunya, Barcelona, Spain, **2** Centro de Investigación Biomèdica en Red en Bioingeniería, Biomateriales y Nanomedicina (CIBER-BBN), Barcelona, Spain

Abstract

In machine olfaction, the design of applications based on gas sensor arrays is highly dependent on the robustness of the signal and data processing algorithms. While the practice of testing the algorithms on public benchmarks is not common in the field, we propose software for performing data simulations in the machine olfaction field by generating parameterized sensor array data. The software is implemented as an R language package chemosensors which is open-access, platform-independent and self-contained. We introduce the concept of a virtual sensor array which can be used as a data generation tool. In this work, we describe the data simulation workflow which basically consists of scenario definition, virtual array parameterization and the generation of sensor array data. We also give examples of the processing of the simulated data as proof of concept for the parameterized sensor array data: the benchmarking of classification algorithms, the evaluation of linear- and non-linear regression algorithms, and the biologically inspired processing of sensor array data. All the results presented were obtained under version 0.7.6 of the chemosensors package whose home page is chemosensors.r-forge.r-project.org.

Citation: Ziyatdinov A, Perera-Lluna A (2014) Data Simulation in Machine Olfaction with the R Package *Chemosensors*. PLoS ONE 9(2): e88839. doi:10.1371/journal.pone.0088839

Editor: Hans A. Kestler, University of Ulm, Germany

Received June 20, 2013; **Accepted** January 13, 2014; **Published** February 26, 2014

Copyright: © 2014 Ziyatdinov, Perera-Lluna. This is an open-access article distributed under the terms of the Creative Commons Attribution License, which permits unrestricted use, distribution, and reproduction in any medium, provided the original author and source are credited.

Funding: This work was funded from the European Community's Seventh Framework Programme (FP7/2007–2013) under grant agreement no. 216916: Biologically inspired computation for chemical sensing (NEUROChem), the Ramon y Cajal program from the Spanish Ministerio de Educación y Ciencia and TEC2010-20886-C02-02. CIBER-BBN is an initiative of the Spanish ISCIII. The funders had no role in study design, data collection and analysis, decision to publish, or preparation of the manuscript.

Competing Interests: The authors have declared that no competing interests exist.

* E-mail: andrey.ziyatdinov@upc.edu

Introduction

Data sharing plays an important role in the fields of computer science, statistics and machine learning. In statistical genetics, The Human Genome Project made the full human genome publicly available on the NCBI website in 2001 [1]. That has been one of the key factors in enabling impressive developments, not only in fields related to biological science, but also in statistical genetics and bioinformatics. The web site of The University of California at Irvine (UCI) Machine Learning Repository is an example of the way the machine learning community sets data repository standards and provides educational resources and open-access benchmarking material. This web site contains over 200 data sets from different theoretical domains, including results from data generators. Simulated data is an option when data collection is complicated by issues related to technological limitations, large problem size, privacy agreements or the time required to gather the data. In statistical genetics, The Genetic Analysis Workshops approach current analytical problems by making both real and simulated data sets available to investigators worldwide. The use of simulated data is a widely accepted practice for evaluating the performance of computer algorithms and can be found in many computer science publications.

The purpose of machine olfaction is to design systems able to recognize smells. An experimental device typically consists of an array of gas sensors, acquisition electronics and a software unit for pattern recognition. Such a device, also known as an *electronic nose*, was originally proposed by G. Dodd and K. Persaud in 1982 [2]. The authors introduced a principle for discrimination among complex odourant mixtures inspired by the way the olfactory system processes the signals from broadly tuned receptor cells. In the work of the authors, it was shown that discrimination between odour classes can be performed by means of an array composed of sensors with overlapping performance profiles, instead of highly specific sensors. Signals recorded from the sensors form a special fingerprint in response to odours, however data processing of such multivariate responses was always a crucial stumbling block in the design of the electronic nose.

The practical application of instruments based on sensor arrays is very sensitive to the robustness of the data processing methods involved [3]. In last three decades, substantial advances have been made in signal and data processing of sensor array data [3–5], including in biomimetic or bio-inspired approches [6,7], although no public repository of data sets has yet been established. The need for a repository of benchmarks has been already mentioned [5], but there are still few data sets publicly available. The UCI Machine Learning Repository contains an archive of 13910 measurements from 16 chemical sensors aimed at tackling the problem of drift compensation in sensor array data [8]. As far as we can ascertain, this is the unique example of an open data set in machine olfaction. We believe that data generators for simulation experiments might be a step forward for the development and testing of data processing algorithms, while the setting up of a data repository and the collection of data sets for this repository would

be a productive long-term activity for the machine olfaction community.

The need for data sets specifically designed for machine olfaction applications arises from the fact that this field has a list of practical problems, which are not common to other machine learning domains. Signals acquired from gas sensors are prone to drift due to the intrinsic instability of sensor devices and environmental changes over the course of the experiment. Any transfer of the applications from the original experimental conditions to a new set up also results in certain instrument re-calibration problems. Scenarios important for testing the application include: sensor replacement and sensor failure (for evaluation the robustness of the array), adaptation and habituation tasks (for design of event-based pattern recognition algorithms), and a number of biologically inspired scenarios such as background suppression (for running neural models to simulate the biological olfactory pathway). Parameterization of the difficulty of each scenario is another important issue for the benchmarking of algorithms designed to address the above problems. For further information on the topic, the reader is referred to the most recent review of signal and data processing in machine olfaction [3] and to the thesis of B. Raman for the introductory material relating to neuromorphic data processing in machine olfaction [9].

The development of the package *chemosensors* was initiated within the framework of the NEUROChem project [10]. The testing of the neuromorphic computational models designed in the project necessitated large scale sensor array data (a large number of sensors in the array) and support for multicomponent gas mixtures. Although neuromorphic simulations were the first application of the generator tool, the simulated data can be used for a general-purpose experiments in machine olfaction. These typically comprise three steps. In the first step, the practitioner considers an experimental scenario. The scenario typically is defined by a list of analytes and their concentrations and the task type, for example, classification or regression. In the second step, transient signals are acquired from the sensors in array. Common practice is to pre-process the signals to compensate for noise and to extract the features relevant for the discrimination task in the specific scenario. In the third step, data analysis relevant to the given scenario is performed. The decisions made in the first step are the most crucial, in the sense that any further improvement is now difficult, if any critical errors were made at the beginning. The *chemosensors* package is mainly focussed on helping the design of a signal processing toolchain by providing the facilities for data simulation. The challenge of this initial step is to find the best possible combination of analytes and sensors which can discriminate between the analytes. Different types of sensors are evaluated by looking at their key response characteristics for the analytes involved in the specific scenario. Typically, the main characteristics of interest are the sensitivity to target analytes, the selectivity to target analytes across the interferents, and the stability of the sensors.

Our *chemosensors* package allows one to parametrically design an array of virtual sensors and to use it as a data generation tool. The simulation of a single sensor is based on a set of physico-chemical models for conducting polymers, which were derived under simplified assumptions and were presented in our earlier work [11], where models emulating different types of noise (including drift) in sensors also were constructed. The software is written in the R language, is organized as a standard package, available on the R-Forge repository and includes installation instructions and code documentation [12,13]. The package presented is aimed at providing an open framework of data simulation to tackle the specific issues in machine olfaction previously mentioned. We propose defining the difficulty level of scenarios as the similarity between gas classes, this is independent of the sensor data or simulation models for data generation.

The R language environment is a widely used framework for the distribution of data sets and software for data generation. Published packages for data simulation include the **fwsim** package for functional magnetic resonance imaging [14], the packages **IBDsim** and **hapsim** in statistical genetics [15,16] and the **simFrame** package for building a general-purpose framework for statistical simulations [17].

Our manuscript is organized as follows. We begin with a description of the materials and methods used to create the *chemosensors* package. Then we explain the parameterization of simulations, and show examples for three machine olfaction tasks: the benchmarking of a classification algorithm, the evaluation of linear and non-linear based regression algorithms and the modelling of the chemotopic convergence of receptor neurons in the early olfactory pathway. Finally, we summarize our work in a Conclusions section.

Materials and Methods

Reference Data Set

The software package includes the simulation models, which were trained with a reference data set as described in [11]. The reference data used in that work (UNIMAN data set) was collected in The University of Manchester (UNIMAN, UK). The long-term measurements of three analytes ammonia, propanoic acid and n-butanol, at different concentration levels, were performed on an array composed of 17 conducting polymer sensors. The measurement protocol implied that sensors were exposed to a rectangular gas pulse of 329 s, and transient signals from the sensors were recorded at 1 Hz sampling frequency. The periodic measurements lasted over 10 months and resulted in 3925 samples stored in the raw data format. Hence, the UNIMAN data set can be represented as a three-dimensional data array of size $3925 \times 329 \times 17$.

The UNIMAN data set is unique, due to the methodology and precision on the gas delivery station jointly with the long-term experiment. The applications on processing of these data are related to scenarios of gas identification complicated by the noise observed in the sensor signals (mainly the long-term drift noise). The detailed information about the UNIMAN data set and list of related applications can be found in [11] and references therein.

Input Protocol

Three different analytes can be used for data simulation, which correspond to the three analytes: ammonia, propanoic acid, and n-butanol in the reference data set. For the sake of simplicity, we use the letters A, B and C to refer to these. Table 1 reports the concentration range for each analyte with concentration units expressed in volume fraction *vol.%*.

The input concentration is defined by a step function, and the lengths of both the exposition and the cleaning phase are equal to 60 time units. This corresponds to the protocol given in the reference data set.

The dynamic range of the virtual sensors is limited to the range from 0.01 to 0.1 vol.% for analytes A and B and to 0.1 to 1 vol.% for analyte C. This corresponds to the range of analyte concentrations in the reference data set given in Table 1.

A transient sensor signal, the output vector $x(t)$, is generated in response to a mixture of analytes, with input concentration matrix $C_0(t)$. The columns of the matrix $C_0(t)$ encode the concentration of three analytes A, B and C. We use i to index the columns of

Table 1. Dynamic range of concentrations for three gases used in the *chemosensors* package.

Gas Label	Analyte	Concentration range, vol.%
A	Ammonia	0.01–0.05
B	Propanoic acid	0.01–0.05
C	n-Butanol	0.1–1

Dynamic range of concentrations for three gases A, B and C, which correspond to three analytes in the reference UNIMAN data set: ammonia, propanoic acid and n-butanol, respectively.
doi:10.1371/journal.pone.0088839.t001

$C_0(t)$, where i takes values 1, 2 and 3. The response of an array of sensors can be expressed as a matrix $X(t)$ comprised of signals from the sensors given in the columns. The number of rows, in both matrices $C_0(t)$ and $X(t)$, is equal to the number of samples per unit time.

Function $C_0(t)$ is defined to be a step function of length 60 time units and the amplitude of the step is denoted by C_0. A time stamp, when the exposition phase ends and the cleaning phase starts, is known as quasi stabilization time and the value of the signal at this point, here x_{ss}, is known as the steady-state value.

Simulation Models

In the *chemosensors* package we used the models designed for polymer based gas sensors and validated these models on the seventeen sensors and three analytes at different concentrations from the UNIMAN data set [11]. This group of models took a matrix of concentrations $C_0(t)$ as input and produced a matrix of sensor array data $X(t)$ as output. Two models, sorption and calibration, emulated the time response of the sensors in the array under noise-free conditions. Three models, concentration noise, sensor noise and drift noise, injected noise to the generated data at different steps of the simulation flow. The response of a single sensor to a mixture of analytes is controlled by the Langmuir isotherm being part of the sorption model. The Langmuir isotherm implies a competitive sorption behaviour and results in a non-linear response to a mixture of analytes. The maximum number of analytes in the mixture is three, as the UNIMAN data set was measured only for three analytes.

The parametrization of the simulation models is summarized in Appendix S1, while the complete description of the models is available in our previous work [17]. Appendix S2 also presents a quantitative comparison between simulated and real data to give the reader the confidence in the data generated by the *chemosensors* package.

Virtual Sensor Array

The simulation models described in Appendix S1 are implemented in the *chemosensors* package as S4 classes in R [12]. The main class of the package SensorArray represents a virtual sensor array and inherits classes from the simulation models, which are SorptionModel, SensorModel, ConcNoiseModel, SensorNoiseModel and DriftNoiseModelf. Table 2 shows the relationship between the simulation models and the classes in the first two columns. The parameters derived from the reference UNIMAN data are stored in the data sets reported in the third column of Table 2. In addition, the data set UNIMANshort contains the short-term reference UNIMAN sub-set of the first 200 samples. All the data sets are distributed with the *chemosensors* package and can be loaded into the R environment by the data function.

Table 2. Organization of simulation models in the *chemosensors* package.

Simulation Model	Class	Data set
Sorption Model	SorptionModel	UNIMANsorption
Calibration Model (steady-state)	SensorModel	UNIMANdistr
Calibration Model (transient)	SensorDynamics	UNIMANtransient
Concentration Noise Model	ConcNoiseModel	–
Sensor Noise Model	SensorNoiseModel	UNIMANsnoise
Drift Model	DriftNoiseModel	UNIMANdnoise

Simulation models, their classes and associated data sets of parameters computed for the seventeen UNIMAN sensors.
doi:10.1371/journal.pone.0088839.t002

In this Section, we describe the basic slots of the SensorArray class and report their relationship to the parameters of the simulation models. Table 3 summarizes the information about the basic slots of SensorArrayclass.

Virtual sensors can be thought as replicas of the 17 UNIMAN sensors. The data sets of the package store parameters related to the simulation models computed for the UNIMAN sensors (See Table 2). When a virtual sensor is initialized, it adopts one of the pre-computed 17 profiles. By means of such model assembly, one can create a virtual sensor array by controlling only two slots of SensorArray class in the basic configuration.

- The num slot represents the types of sensors in the array. It is an integer vector whose length is equal to the number of sensors in the array. The elements of the vector num can take values from 1 to 17, corresponding to one of the seventeen sets of parameters derived from the UNIMAN sensors. These parameters include K_i, $\beta_{i,k}$, $\tau_{1,i}$, and $\tau_{2,i}$ as presented in Appendix S1.
- The nsensors slot stores the number of the sensors in the array.

Table 3. Basic slots of SensorArray class in *chemosensors* package.

Slot	Default Value	Range of values	Short Description
num	1:2	1, 2, … 17	type of sensors
nsensors	2	1, 2, …	number of sensors
ngases	3	1, 2, 3	number of gases
gnames	c('A', 'B', 'C')	any strings	names of gases
concUnits	'perc'	supported string	concentration units
alpha	2.25	>0	sensor non-linearity
beta	2	≥ 0	sensor diversity
csd	0.1	≥ 0	concentration noise sd
ssd	0.1	≥ 0	sensor noise sd
dsd	0.1	≥ 0	drift noise sd
ndcomp	1	1, 2, 3	number of drift components
ndvar	0.86	[0, 1]	importance of drift components
tunit	1	1, 2, …	length of a gas pulse

Description of basic slots of SensorArray class necessary to parameterize a virtual sensor array.
doi:10.1371/journal.pone.0088839.t003

For instance, a virtual array created with parameters num 1:2 and nsensors 2 has two sensors that represent the first two sensors in the UNIMAN data set. That two UNIMAN sensors were different by the polymer material the film of the sensors was composed from, and the sensors had different chemical selectivity and sensitivity characteristics in response to the three examined analytes: ammonia, propanoic acid, and n-butanol. The two virtual sensors possess the same relationships from the UNIMAN sensors, which are expressed in the parameters of the simulation models, please see [11] for further details.

If one needs an advanced configuration of the array, other slots of SensorArray class are available. Many slots are implemented as easy-to-use scaling factors.

- The alpha slot is a scaling factor for controlling the non-linearity of a sensor. If alpha is equal to 1, then the scaling is omitted and the virtual sensors take the sorption affinities K_i from the UNIMANsorption data set according to their types (slot num). If alpha is not equal to 1, then the magnitudes of the affinity coefficients K_i are scaled up (alpha >1) or scaled down (alpha <1) proportionally, so that the relative relationship along the seventeen sorption profiles is preserved. Non-linearity in a sensor increases with an increase in alpha, this is a consequence of the fact that sensors under the Langmuir relation in the sorption model tend to a non-linear behaviour when the coefficients K_i are large. The value of zero is not allowed, because then the sorption model given in Equation (1) in Appendix S1 would be meaningless.

  Another role of the scaling operation by alpha is the regulation of a response to a mixture of analytes. As the output of the sorption model is a weighted (or penalized) sum of the inputs, more penalization is induced with greater magnitudes of K_i and, thus, a greater value of alpha. The default value of the slot (2.25) has been selected to favour a more balanced penalization of sensors' responses to different mixtures of the three analytes.

- The beta slot is a scaling factor for controlling the diversity across sensors in the array. If beta is equal to 0, then the scaling is omitted and the sensitivity coefficients $\beta_{i,k}$ in the calibration model of virtual sensors are taken from the coefficients estimated for the UNIMAN sensors. If beta is greater than 0, than the coefficients $\beta_{i,k}$ are derived from the uniform distributions with parameters stored in UNIMANdistr data set. The value of beta defines the spread of the distributions. The diversity across sensors increases with an increase in beta. The default value of beta (2) corresponds to a moderate level of diversity.

Note that one can create a copy of the UNIMAN array of the seventeen sensors under the simulation models by setting up alpha to 1 and beta to 0. Thus, the virtual array will replicate the same properties of non-linearity and diversity as the UNIMAN array.

The magnitude of noise generated by the simulation models is mainly controlled by three scaling slots csd, ssd and dsd, which correspond to concentration, sensor and drift noise models respectively. Values of csd, ssd and dsd typically range from 0 to 1. A value 0 implies a noise-free mode, and the value of 1 has been selected to correspond to the level of noise observed in the reference UNIMAN data set. The default values of the three slots are equal to 0.1, which supposes a moderate level of noise.

- The csd slot is a scaling factor for controlling the concentration noise. It scales the covariance matrix Σ_c in the concentration noise model. The default value is 0.1.
- The ssd slot is a scaling factor for controlling the sensor noise. It scales all the covariance matrices $\sigma_{i,k}$ in the sensor noise model. The default value is 0.1.
- The dcsd slot is a scaling factor for controlling the drift noise. It scales the covariance matrix Σ_d in the drift noise model. The default value is 0.1.
- The ndcomp slot encodes the number of drift components. Its value is equal to the number of columns in the matrix P of the drift noise model. The default value is 1. This corresponds to the one drift component which has been observed in the reference UNIMAN sensor array data [18]. The slot can possess the values 1, 2 or 3.
- The ndvar slot defines the structure of the drift noise and encodes the importance of drift components. The slot is a vector which contains the diagonal elements of the covariance matrix Σ_d of the drift noise model. The values of the elements in ndvar vector lie in the range [0,1]. The default value is 0.86, given that the value of ndcomp slot is 1. The slot can be a vector of up to 3 elements, as limited by the ndcomp slot. If three drift components are given, then the default values of ndvar are 0.86 0.06 and 0.05.

Workflow

The workflow of data simulations in the *chemosensors* package consists of several steps. In the first step, the practitioner defines analytes and concentration levels for a scenario and the sensors required to build an appropriate array. The basic initialization parameters to build a virtual array include the sensor types num and the number of sensors nsensors (along with others for more advance configurations). The package contains a special class Scenario for the representation of analytes and concentrations. The plot methods of the SensorArray class have been designed to perform the exploratory data analysis on the sensor array data.

In the second step, the practitioner generates sensor array data by a single command. In particular, the predict method of the SensorArray class takes as input a matrix of analyte concentrations and returns as output a matrix of sensor array responses. Parallelized computation of sensor signals is supported, this is necessary in the case of long-term scenario or a large number of sensor elements.

In the third step, the practitioner performs a data analysis on the sensor array data by means of any convenient software tool. In general, the software for data analysis can be an external program, and both matrices of concentrations and sensor signals can be easily exported in a format like *csv* by standard R facilities, as no specific data format is assumed in the package.

The noise level in the array is a simulation parameter which can be updated on-the-fly in the simulation. We consider such flexibility in controlling noise to be a useful option, when the performance of a specific sensor is evaluated under drift-free conditions or when the level of noise is a parameter in benchmarking data analysis algorithms.

Installation

The source code of the *chemosensors* package is hosted on the R-Forge web page [13,19]. The package is also available on the official CRAN repository of the R packages and can be installed by typing the following command in R:

install.packages("chemosensors").

That will install the latest stable version of the package and all its dependencies from the CRAN repository. The distributed package is platform-independent and self-contained.

Results

The *chemosensors* package is organized around the S4 classes of simulation models (See Table 2), and the implementation of the classes shares some common features.

- Class constructors can be called in the standard form for S4 classes using the new function. For the sake of simplicity, every class has a function, which serves as a wrapper for the class constructor and has the same name as the class.
- The standard methods show, print and plot have been designed for all classes, this makes the output more verbose.
- One uses @ to access slots of a S4 object. Special *get* and *set* methods have been implemented to access most slots of the simulation models, and the methods have the same names as the slots.

The following code shows a quick-start example of a simulation, where one defines a custom matrix of concentrations, creates a sensor array and generates the data. This is an example of the regression scenario of one single gas A given at several concentration values.

```
conc <- matrix(0, nrow = 120 * 3, ncol = 3)
conc[61:120, 1] <- 0.01
conc[181:240, 1] <- 0.02
conc[301:360, 1] <- 0.05
```

```
sa <- SensorArray(num=1:4, tunit = 60)
sdata <- predict(sa, conc)
```

The concentration matrix conc encodes three pulses of analyte A at different concentrations 0.01, 0.02 and 0.05%. vol. The array sa is composed of four sensors of four different sensor types, and the tunit parameter is set to 60 to enable the sensor dynamic model for pulses with step 60. Each gas pulses consists of two parts of equal length 60, the gas exposition phase and the cleaning phase (the gap between two consequent exposition phases). Figure 1 (a) depicts the change in analyte concentrations over time, and Figure 1 (b) depicts the signals from the four sensors in response to the concentrations. One can suppress the drift noise in the array by setting the dsd slot to zero and repeat the simulation, as shown in the code below. Figure 1 (c) depicts the sensor signals under drift-free conditions.

```
dsd(sa) <- 0
sdata <- predict(sa, conc)
```

In this section, we present some examples of the use of the *chemosensors* package. Firstly, we introduce some basic topics related to the use of the Scenario class, the configuration of a sensor array and the generation of sensor array data. Secondly, we give examples of data analysis performed on the simulated data produced by the package. In particular, we show examples of benchmarking a classification algorithm, the evaluation of two regression algorithms and some biologically-inspired modelling.

To perform the classification and regression analyses we use the *caret* package developed by Max Kuhn [20]. This package provides a unified workflow for the process of constructing a predictive model with the support of automated tools for data pre-processing, resampling procedures, feature selection and model tuning. We also use Self-Organizing Maps (SOM) as implemented in the *kohonen* package for some biologically-inspired modelling [21].

Defining Scenarios

The Scenario class has been introduced to serve as a more compact representation of a concentration matrix. The labels of analytes and the length of pulses are the main parameters required to specify a scenario. For instance, the conc matrix in the previous example can be alternatively constructed by creating an object of the Scenario class and applying the getConc method to extract a concentration matrix, as shown in the code below.

```
sc <- Scenario(c(''A 0.01'', ''A 0.02'', ''A
0.05''), tunit=60)
conc <- getConc(sc)
```

The Scenario class also encodes a training set and a validation set (or test set) at the time of initialization. The parameters T and nT respectively encode gas labels and the number of samples per label for the training set, and the parameters V and nV also obtain for the validation set. The training set is followed by a validation set, as is typically accepted in machine olfaction experiments. Randomization of the samples is controlled by the logical parameter randomize. One can re-create the previously created sc scenario by specifying more parameters, as shown in the following code.

```
sc <- Scenario(name=''Regression'', tunit=60,
concUnits=''perc'',
  T=c(''A 0.01'', ''A 0.02'', ''A 0.05''),
   nT=30,
  V=c(''A 0.01'', ''A 0.02'', ''A 0.05''),
   nV=30,
  randomize=TRUE)
```

```
sc
```

```
> Scenario 'Regression' of 180 samples, tunit
60, randomize TRUE
> - gases A, B, C
> - Training Set: A 0.01 (30), A 0.02 (30), A 0.05
(30)
> - Validation Set: A 0.01 (30), A 0.02 (30), A
0.05 (30)
```

The show method prints the basic information about sc object. The plot method provides the same information by depicting the unique gas labels in the training and validation sets. Figure 2 shows the graphics produced by the plot method for the scenario object sc showed above.

```
plot(sc)
```

The resulting scenario sc represents a regression problem for analyte A given at three concentrations 0.01, 0.02 and 0.05. In both training and validation sets there are 30 samples per concentration. It may sometimes be necessary to update a scenario once it is initialized. In the code given below, the add method is used to supplement the training set with two more gas labels; this might improve the accuracy of the model because of a more representative set of concentrations.

```
add(sc) <- list(''A'', 0.03, 30, ''T'')
add(sc) <- list(''A'', 0.04, 30, ''T'')
```

In practice, it might be necessary to retrieve extra data from the scenario in addition to the matrix of concentrations. The sdata.frame method returns a data frame with additional columns

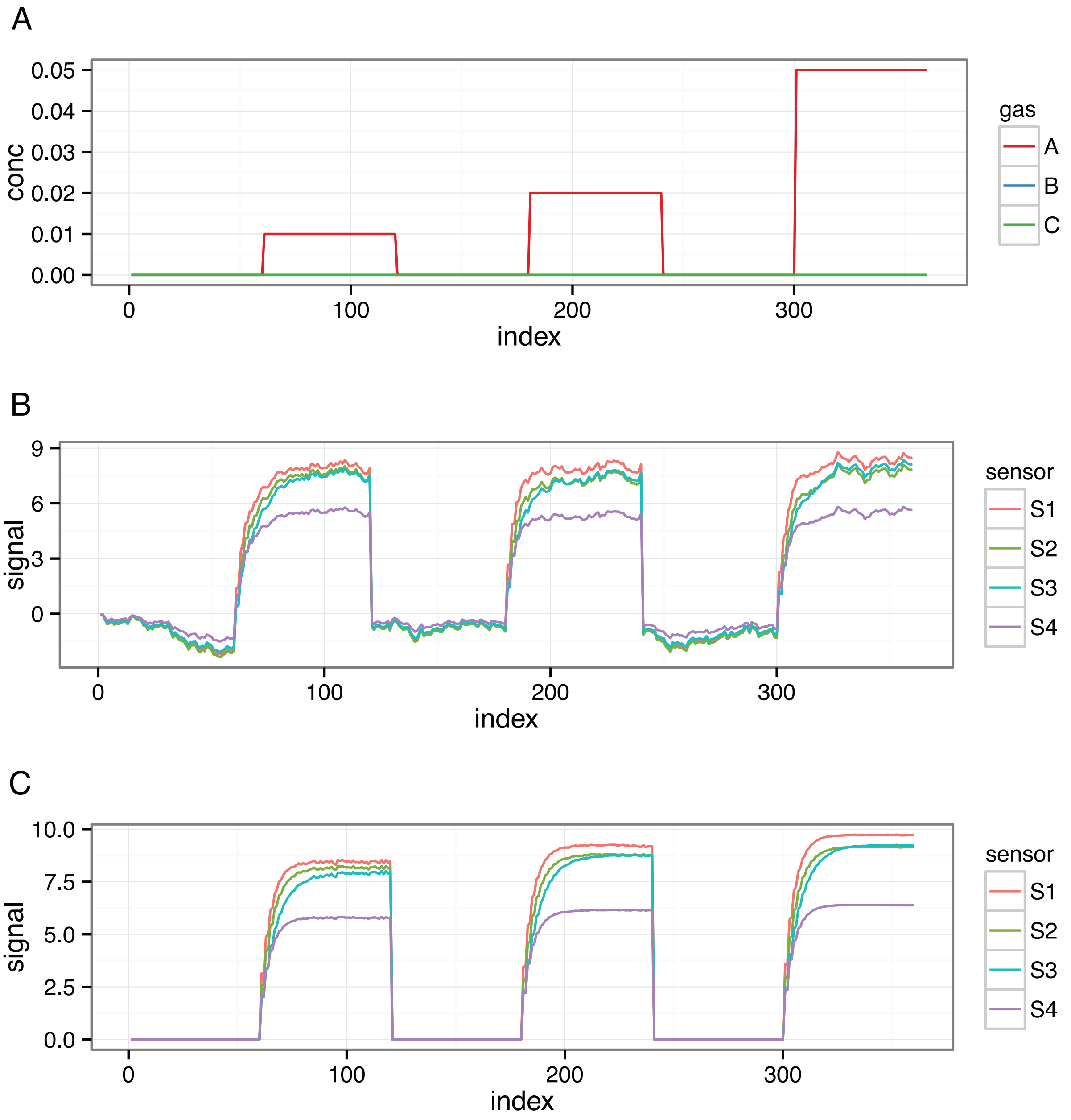

Figure 1. Matrices of analyte concentrations and sensor signals in a simulation with a virtual array of four sensors. On the X axis of each panel, the index values correspond to the row index in the two input concentration and output sensor data matrices of the data generator. Consequently, the values in the columns of these matrices are plotted jointly on the Y axis, while the legend on the right annotates the column names. Panel (a) shows three pulses of analyte A at three different concentrations 0.01, 0.02 and 0.05 vol.%, while the concentration of the other two analytes B and C are at zero level. Panel (b) shows transient signals of four sensors labelled as S1, S2, S3 and S4 in response to the pulses from Panel (a) when all three noises in the sensor array are set up at the 0.1 level. Panel (c) shows sensor signals in response to the pulses under drift-free conditions, while the other two concentration and sensor noises are remained at the 0.1 level. The signals allow for a visual discrimination between the three pulses.
doi:10.1371/journal.pone.0088839.g001

which represent gas labels, time units and set index (training or validation set). In the code given below, the sdata.frame method is applied to the regression scenario created above, and samples indexed from 58 to 62 are printed.

```
cf <- sdata.frame(sc)
cf[58:62, ]
```

```
> index A B C glab lab tpoint time set
>58 1 0.00 0 0 Air Air air 58 T
>59 1 0.00 0 0 Air Air air 59 T
>60 1 0.00 0 0 Air Air airout 60 T
>61 1 0.01 0 0 A A0.01 gasin 61 T
>62 1 0.01 0 0 A A0.01 gas 62 T
```

The resulting cf data frame contains both air and gas A labels in the 6th column lab, because every label entry, for example A0.01, in either training or validation set encodes a gas pulse consisting of two parts, the exposition phase of the length tunit and the cleaning phase of the same length tunit. Note that the cf data frame has a

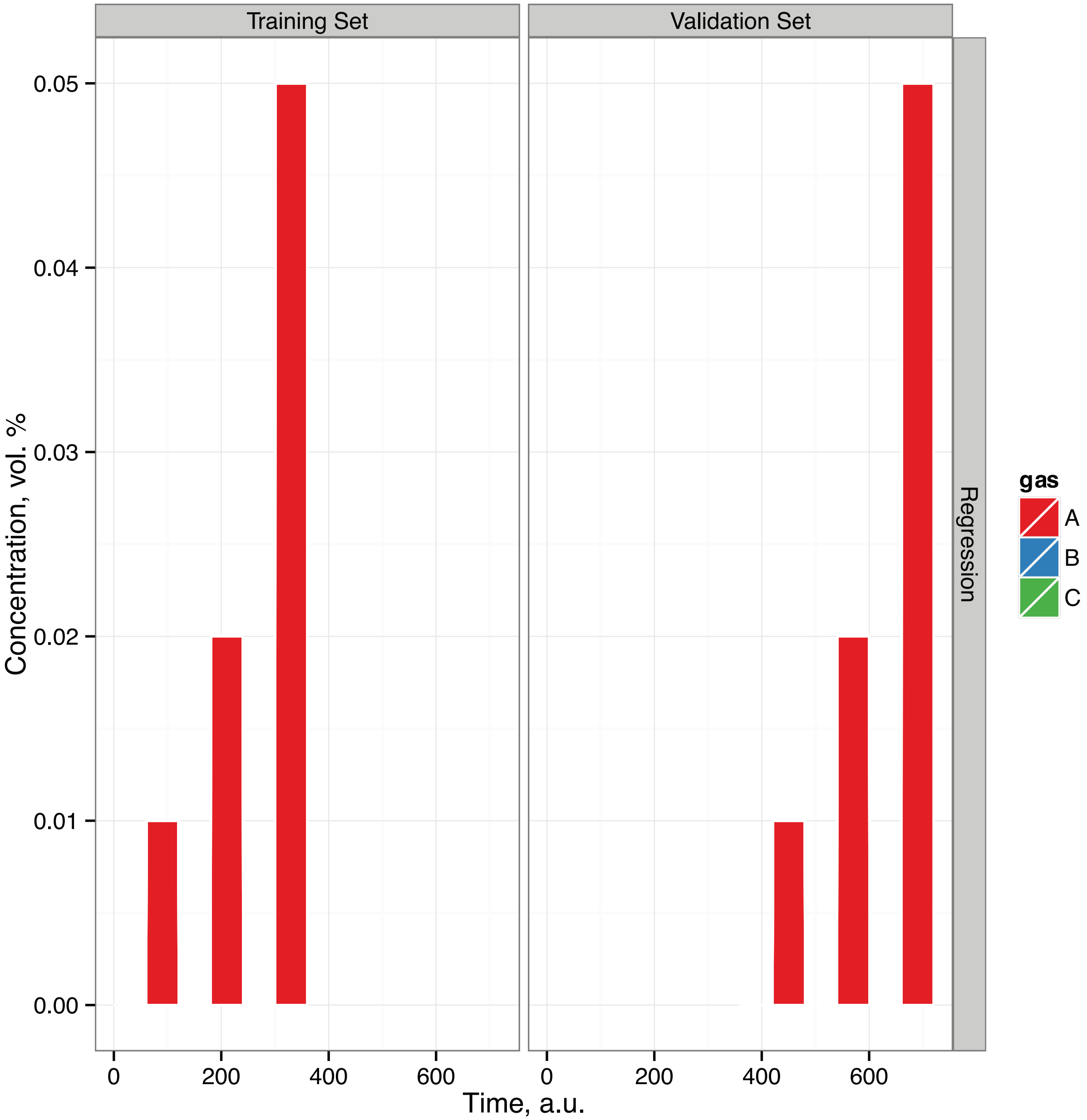

Figure 2. Plot showing the training and validation set, product of the plot method applied to a regression scenario. The scenario is defined as a regression on analyte A with both training and validation sets consisting of three pulses of concentrations of 0.01, 0.02 and 0.05 vol.%. The plot method applied to a scenario object shows only the unique labels given at training and validation sets. One can apply the show method to a scenario object to get more detailed information.
doi:10.1371/journal.pone.0088839.g002

special column tpoint for encoding events on changes between the exposition and cleaning phases of the gas pulse. This variable takes values air, airin, airout, gas, gasin and gasout, and is used for transient feature extraction from transient sensor signals.

- transient feature: All samples are used.
- steady-state (alias ss) feature: Samples with tpoint labels equal to gasout are extracted, this corresponds to the time stamp when the exposition phase is finished and the cleaning phase is to be started.
- step feature: The same samples as for steady-state feature are used, but the sensor data with tpoint labels equal to airout are subtracted. This method of feature extraction also reduces the drift noise.

For example, the concentration matrix depicted on Figure 1 (a) has three time stamps of gasout at 120, 240 and 360 time units, which correspond to the time of extraction of the steady-state signal.

Ten scenarios for machine olfaction proposed in the framework of the NEUROChem project [10] are given File S1. The document contains the description of each scenario in terms of training and validation sets, definition of scenario difficulty and the R code to create an object of Scenario class.

Configuring Sensor Array

From now on, we will use the default value 1 of the tunit parameter to create any virtual sensor array. Such parametrization means the only steady-state feature in the sensor response, instead of, for example, 120 transient features in the case of the tunit parameter equal to 60. This strategy seems to be reasonable, as that allows us to significantly reduce the number of samples needed to be simulated for testing pattern recognition models, while we will exploit one the most commonly used features from the transient sensor response (steady-state). Hence, the input for the simulation models will be trivial gas pulses each parametrized with tunit 1, that results in one sample of a gas in the exposition phase and one sample of the air in the cleaning phase. The response to the air sample represents a baseline level in the signal, which typically is subtracted from the response to the gas sample, being a standard drift-correction method in the stage of the signal processing (that corresponds to the feature parameter equal to step in the sdata.frame method).

There are several ways to configure a virtual sensor array in the *chemosensors* package. Basic selection of sensor types is controlled by num parameter among other parameters. Information stored in the data sets given in Table 2 characterize the UNIMAN sensors (or sensor prototypes) and can be used for the selection of particular sensor types. The SensorArray class has a group of plot methods plotPolar, plotPCA, plotBox and plotResponse for a visual representation of the relation between analytes and sensors. Here, we show an example of a configuration of a sensor array targeted at discriminating between a set of gas classes: pure analytes A and C at different concentrations and binary mixtures of them.

```
set.AC <- c(''A 0.01'', ''A 0.05'', ''C 0.1'',
''C 1'', ''A 0.01, C 0.1'', ''A 0.05, C 1'')
```

The affinity coefficients K_i in the sorption model are important sensor characteristics for the discrimination task posed. The code given below shows how one creates an array composed of all the 17 sensor types and gets the coefficients K_i by the coefficients method.

```
sa <- SensorArray(num = 1:17)
coef <- coefficients(sa, ''SorptionModel'')
str(coef)
```

```
> num [1:3, 1:17] 53.1 43.2 136 65.2 44.1 ...
> - attr (*, ''dimnames'') = List of 2
>   . .$ : chr [1:3]  ''A'' ''B'' ''C''
>   . .$ : chr [1:17]  ''1'' ''2'' ''3'' ''4'' ...
```

The relative importance of the sorption coefficients for analytes A and C is estimated by the following code.

```
sort(coef[''A'', ]/coef[''C'', ])
```

```
>     2      3      1      5      6     16      9
8     4     15
>0.3752  0.3809  0.3906  0.4085  0.6864  0.8087
0.8308  1.1584  1.2877  1.3308
>   10     11     12     13      7     14     17
>1.3837  1.3980  1.7380  2.1962  2.3077  3.6603
6.3235
```

The same comparison can be performed by looking at pre-computed sorption coefficients for the seventeen UNIMAN sensors and stored in the data set UNIMANsorption.

```
str(UNIMANsorption)
```

```
> List of 1
>  $ qkc: num [1:17, 1:3, 1:4] 10.02 9.51 9.52
6.57 9.19 ...
>   . . - attr (*,  ''dimnames'') = List of 3
>   . .  . . $ : chr [1:17] ''1'' ''2'' ''3'' ''4''
...
>   . .  . . $ : chr [1:3] ''A'' ''B'' ''C''
>   . .  . . $ : chr [1:4] ''Q'' ''K'' ''KCmin''
''KCmax''
```

```
K <- UNIMANsorption$qkc[, , ''K'']
sort(K[, ''A'']/K[, ''C''])
```

```
>    2     3     1     5     6    16     9
8    4    15
>0.4307  0.4526  0.4581  0.4820  0.7187  0.8278
0.8666   1.1213   1.2308   1.2881
>   10    11    12     7    13    14    17
>1.2933  1.3123  1.6083  2.0277  2.0775  3.1035
5.0328
```

The order of sensors is slightly different, as sensors in a virtual array are not exact copies of the UNIMAN sensors, but replicas derived from the UNIMAN parameters.

Now we create three different arrays composed of sensors which are different in affinities to analytes A and C. All the arrays are configured to have 12 sensor elements and zero level of the drift noise.

```
sa1<-   SensorArray(num = 1:3,   nsensors = 12,
dsd = 0)
sa2<-  SensorArray(num = c(13, 14, 17), nsen-
sors = 12, dsd = 0)
sa3<-  SensorArray(num = c(1:3,  13,  14,  17),
nsensors = 12, dsd = 0)
```

Arrays sa1 and sa2 include sensors having greater affinity to analyte C and A, respectively. The last array sa3 is composed of sensor types present in both previous arrays.

Principal component analysis (PCA) is one the most widely used shrinkage methods to represent sensor array data in a low-dimensional space [3,5]. Principal components, as data projections, are mutually uncorrelated and ordered in variance. It is well known that the principal components of a data set provide a sequence of best linear approximations to that data [22]. We use the PCA technique to evaluate sensor arrays sa1, sa2 and sa3 in response to a set of gas labels set.AC. In particular, we plot the PCA scores of data projected onto the first two principal components.

The *chemosensors* package contains a list of plot methods suitable for evaluating sensor arrays on a set of analytes by means of exploratory graphics. The plot methods are applied to objects of the SensorArray class, the input is either a concentration matrix or a set of gas labels, sensor array data are generated on the fly, and feature selection from sensor transients is parameterized.

- plotPolar method (default): Sensor array data are computed for a given concentration matrix or a set of gas labels and are

plotted in polar coordinates, where sensor numbers are angles and sensor signals are radii.

- plotPCA method: A principal component analysis (PCA) is computed on sensor array data, and the graphics show a plot of scores on the first two principal components. The percentage of data variance captured by components also is presented.
- plotBox method: Sensor array data are grouped according to gas labels and are shown as a box plot.
- plotResponse method: Both input concentration matrix and output sensor array data, given for a sensor array object, are plotted over time as lines.

All the plot methods share the same list of parameters.

- x: an object of the SensorArray class.
- conc: a matrix of analyte concentrations.
- sdata: a matrix of sensor data in response to a matrix of concentrations conc.
- set: a set of gas labels, which is a parameter alternative to conc (a further concentration matrix is created via Scenario class).
- feature (default value transient): the name of a method for transient feature extraction from sensor array data.
- air (default value FALSE): a boolean value as to whether air samples are to be included or not.

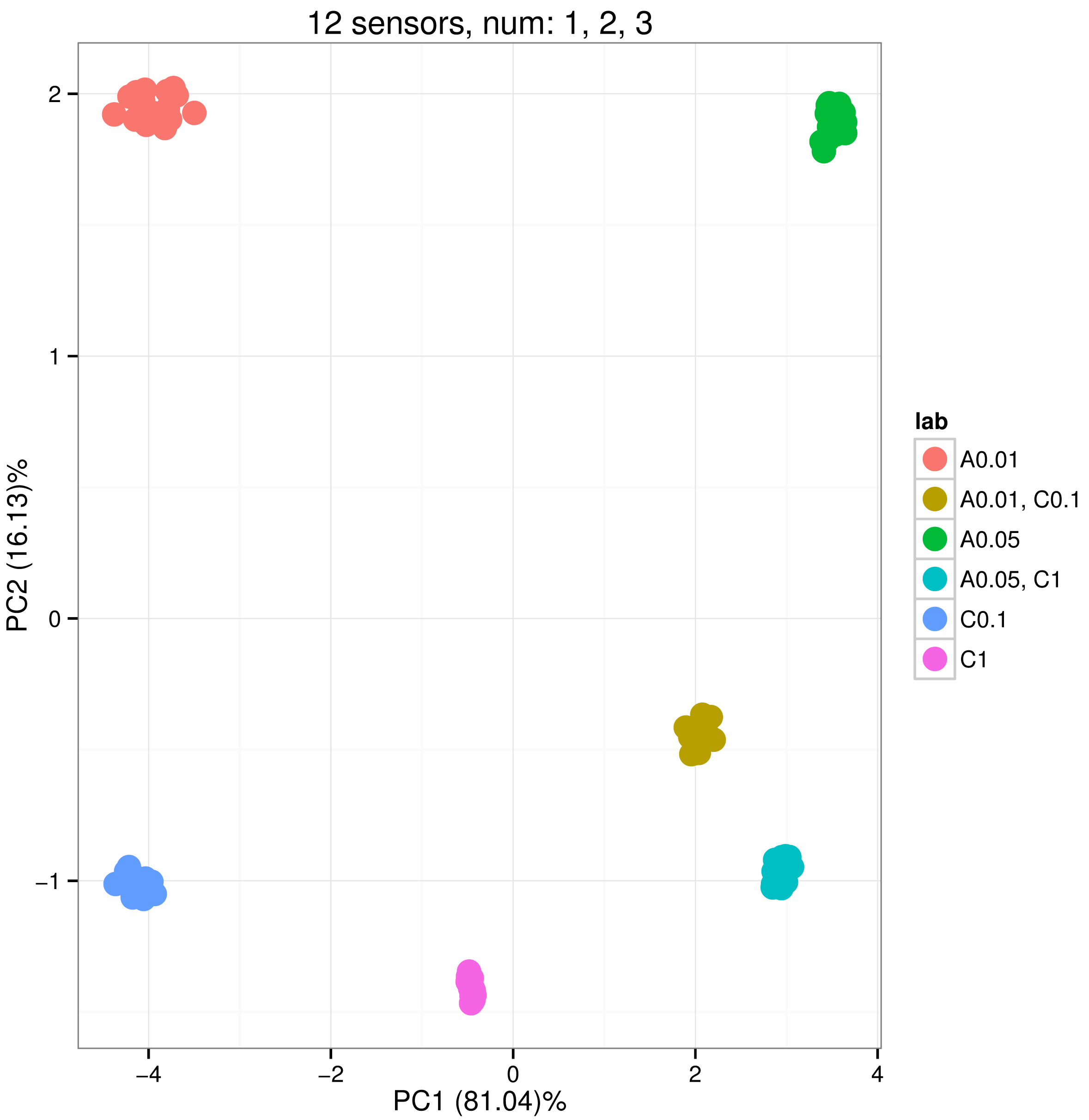

Figure 3. Scoreplot corresponding to the Principal Component Analysis of the sensor array data gathered from the array consisting of 12 sensors of types 1, 2 and 3. The array was exposed to six gas classes: pure analyte A at concentrations 0.01 and 0.05 (labels A 0.01 and A 0.05), pure analyte C at concentrations 0.1 and 1 (C 0.1 and C 1), and two binary mixtures of A and C (A 0.01, C 0.1 and A 0.05, C 1). The concentrations were given at volume fraction units *vol.%*, and the measurement of each gas class was repeated 10 times. The distribution of the scores shows that the sensors in array have more affinity to analyte C that to analyte A. The plot is produced by the plotPCA method applied to the sensor array.
doi:10.1371/journal.pone.0088839.g003

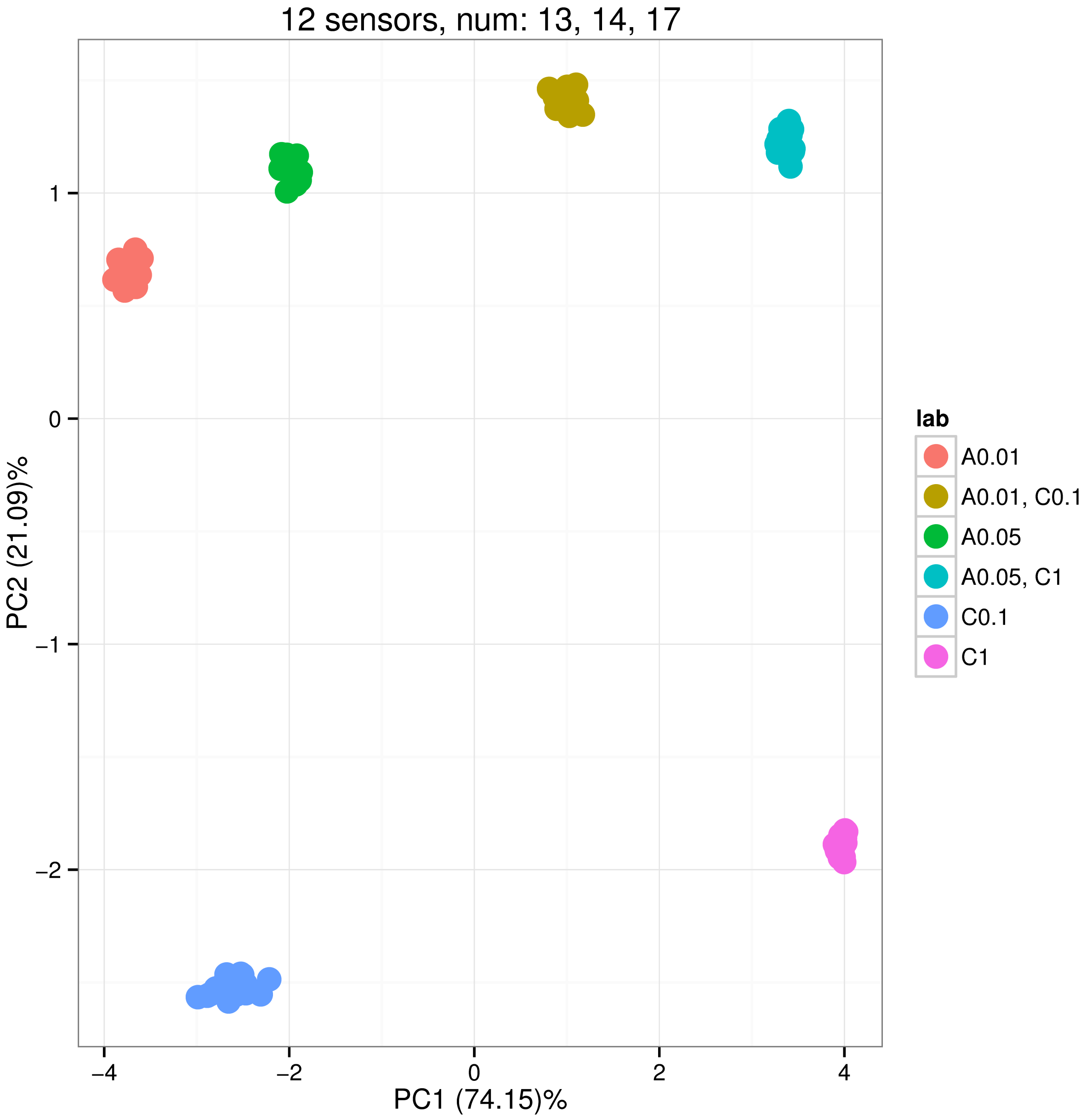

Figure 4. Scoreplot corresponding to the Principal Component Analysis of the sensor array data gathered from the array consisting of 12 sensors of types 13, 14 and 17. The array was exposed to six gas classes: pure analyte A at concentrations 0.01 and 0.05 (labels A 0.01 and A 0.05), pure analyte C at concentrations 0.1 and 1 (C 0.1 and C 1), and two binary mixtures of A and C (A 0.01, C 0.1 and A 0.05, C 1). The concentrations were given at volume fraction units *vol.%*, and the measurement of each gas class was repeated 10 times. The distribution of the scores shows that the sensors in the array have more affinity to analyte A than to analyte C. The plot is produced by the plotPCA method applied to the sensor array.
doi:10.1371/journal.pone.0088839.g004

- gcol (default value FALSE): a boolean value as to whether colours for gas labels are to be computed with the method gcol.

Now we apply the plotPCA method to three sensor arrays sa1, sa2 and sa3 in response to the set of gas labels set.AC.

```
plotPCA(sa1, set=rep(set.AC, 10), air=FALSE)
plotPCA(sa2, set=rep(set.AC, 10), air=FALSE)
plotPCA(sa3, set=rep(set.AC, 10), air=FALSE)
```

We induce 10 repetitions for each gas label and exclude samples of the air in the PCA plot. The default transient feature extraction transient is appropriate for the analysis, as the drift noise was set to zero level when creating the arrays of sensors.

Figures 3, 4 and 5 show the distribution of PCA scores for the three arrays. In Figure 3 the scores of two groups for binary mixtures A 0.01, C 0.1 and A 0.05, C 1 are closer to the scores of groups for pure analyte C; this means that sensors of the sa1 array tend to have a greater affinity for analyte C. On the contrary, Figure 4 shows that sensors of the sa2 array have greater affinity for analyte A. The horizontal line PC2 = 0 can be used to visually pick up such kinds of observations. Figure 5 shows a balanced distribution of classes in terms of affinities for analytes A and C. In addition, this plot shows more diversity in the PCA scores for sa3 array; this can be noted by looking at the amount of variance captured by the two principal components PC1 and PC2 (labels on x and y axis).

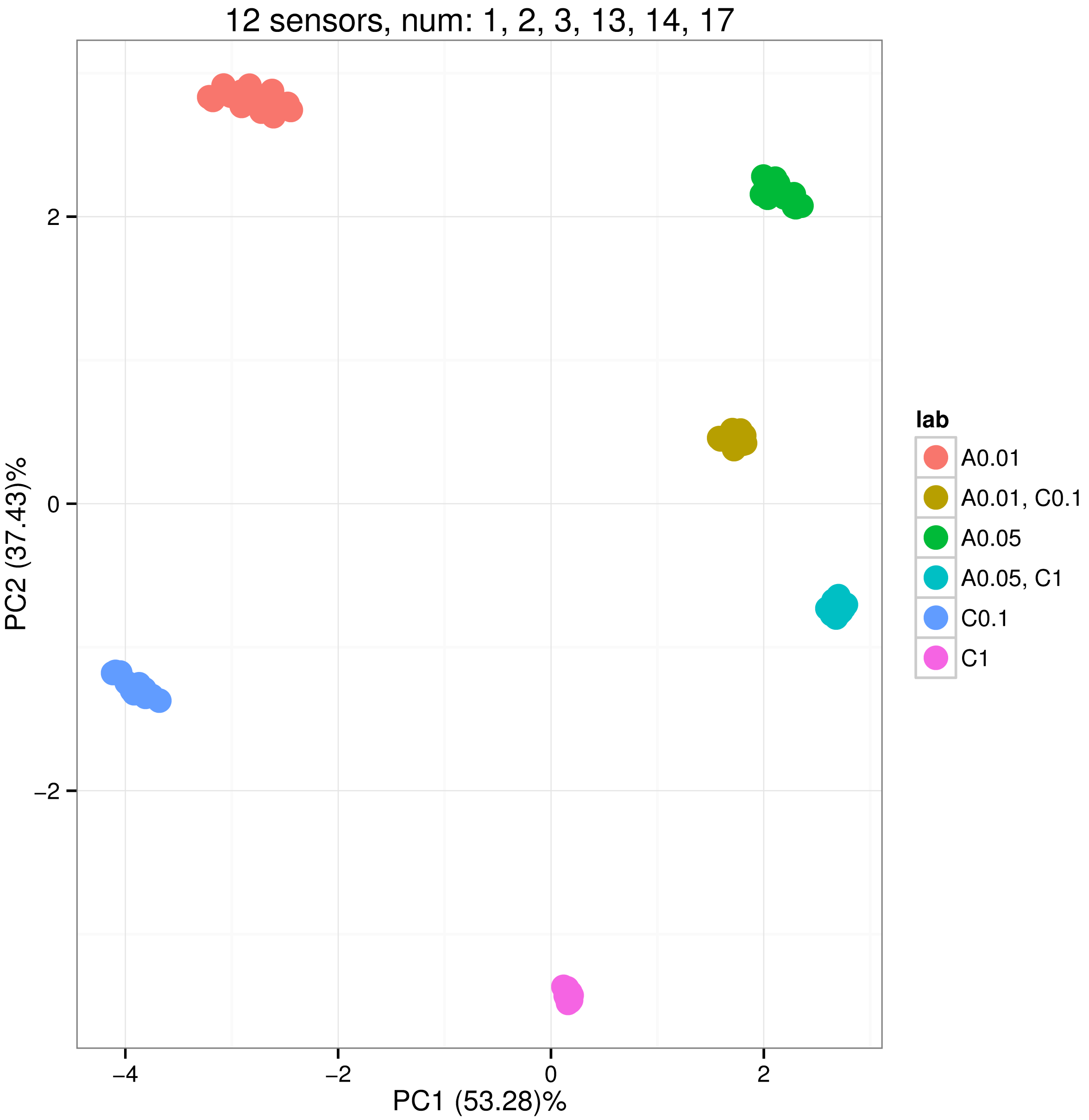

Figure 5. Scoreplot corresponding to the Principal Component Analysis of the sensor array data gathered from the array consisting of 12 sensors of types 1, 2, 3, 13, 14 and 17. The array was exposed to six gas classes: pure analyte A at concentrations 0.01 and 0.05 (labels A 0.01 and A 0.05), pure analyte C at concentrations 0.1 and 1 (C 0.1 and C 1), and two binary mixtures of A and C (A 0.01, C 0.1 and A 0.05, C 1). The concentrations were given at volume fraction units *vol.%*, and the measurement of each gas class was repeated 10 times. The distribution of the scores shows that the sensors in array are balanced in terms of affinity to analytes A and C. The plot is produced by the plotPCA method applied to the sensor array.
doi:10.1371/journal.pone.0088839.g005

Generating Data

Data generation is performed when one has defined a matrix of analyte concentrations and a sensor array. The predict method of SensorArray class takes as input the sa object of SensorArray class and the conc concentration matrix and produces as output the sdata matrix of sensor signals. Typically, data generation is accomplished by running a single command, as shown in the following code.

```
sdata <- predict(sa, conc)
```

To parallelize the computation, one passes the cores (alias nclusters) parameter to the predict method. For example, two cores are specified in the code example given below.

```
sdata <- predict(sa, conc, cores = 2)
```

Another way to configure the computation on several cores is by using the options command, as shown in the following code.

```
options(cores = 2)
```

The are several facilities available in the *chemosensors* package to process the data stored in the conc and sdata matrices. The Scenario class automates the process of creation of concentration matrices. In particular, the getConc method returns a concentration matrix encoded by an object of Scenario class, and the sdata.method method allows the retrieval of such additional

Table 4. Classification performance on scenarios given at three different difficulty levels.

Difficulty	Class 1	Class 2	k	Acc. (train)	Acc. (test)
1	A 0.02	C 0.5	3	1.00	1.00
2	A 0.01, C 0.6	A 0.03, C 0.4	5	0.99	0.94
3	A 0.015, C 0.55	A 0.025, C 0.45	7	0.86	0.74

The k-nearest neighbors algorithm was tested on three two-class classification scenarios at three difficulty levels. The scenario difficulty was defined as the similarity between two gas classes. The classification model was trained under 10-fold cross-validation procedure with 10 repetitions, and the best value of the k parameter was estimated along possible values 3, 5, 7 and 9 for each classification model. The accuracy in prediction of class labels was used to score the models. The model complexity, expressed in value of parameters k, is observed to increase with greater scenario difficulty. The first model provides a perfect performance with a 100% rate of classification, while the last model displays poor accuracy with a classification rate of 0.74 on the test set.
doi:10.1371/journal.pone.0088839.t004

variables as set and tpoint for separation into training and validation sets and for parameterization of transient feature extraction, respectively. The same method sdata.frame applied to an object of the SensorArray class takes as input four basic parameters: an sa object of the SensorArray class, the conc concentration matrix or a cf data frame (obtained from an object of Scenario class by the sdata.frame method), the sdata matrix of sensor signals and the feature parameter to define a method for feature extraction. The following code shows an example of using the sdata.frame method to construct the df data frame, which contains both concentration- and sensor-related information.

```
df <- sdata.frame(sa, conc = conc, sdata = sdata, feature = “step”)
```

Benchmarking of a Classification Algorithm

In this Section, we present a procedure for benchmarking a particular classification algorithm to discriminate a set of gas classes. How one defines the difficulty of the scenarios used for testing is important. Since the level of difficulty has to be independent of the sensor data or simulation models for data generation, we propose determining the difficulty of a scenario by the similarity between analytes in mixture. Such a definition is possible, as the simulation models in *chemosensors* package support mixtures of analytes.

We will use only two classes in the scenarios, constructed as mixtures of two analytes A and C. The first three columns in Table 4 present three scenarios at different difficulty levels. We apply the k-nearest neighbors (KNN) algorithm for classification. It is known that predictions of this method are often accurate, but can be unstable [22]. Thus, we will perform a 10-fold cross-validation procedure (10 repetitions) for the selection of the best parameter k on the training stage with a sufficient number of samples.

In the first step, we generate the gas labels and sensor array data with the *chemosensors* package. We will construct an array based on 17 sensors from all sensor types, and the noise level of all three types will be set to 1. The code below shows an example of producing a data frame df for a scenario of difficulty 1. The size of both the training and validation (or test) set has been selected so that each gas label is represented by 100 samples. This results in 10 samples per fold in the 10-fold cross-validation at the time of the model training.

```
set <- c(‘‘A 0.02’’, ‘‘C 0.5’’)

sc <- Scenario(T = set, nT = 100, V = set, nV = 100,
randomize = TRUE)
conc <- getConc(sc)
cf <- sdata.frame(sc)

sa <- SensorArray(num = 1:17, csd = 1, ssd = 1,
dsd = 1)
sdata <- predict(sa, conc = conc, cores = 2)
df <- sdata.frame(sa, cf = cf, sdata = sdata,
feature = ‘‘step’’)
```

In the second step, we train a model based on the KNN algorithm with the **caret** package. For model tuning, we will explore values 3, 5, 7 and 9 of the parameter k. PCA will be applied for pre-processing of sensor array data; this is one of the common options for building predictive models in machine olfaction [3]. Separation of the training and validation (testing) set will be controlled by the variable lab in data frame df.

```
Xt <- as.matrix(subset(df, set = = ‘‘T’’, se-
lect = snames(sa)))
Xv <- as.matrix(subset(df, set = = ‘‘V’’, se-
lect = snames(sa)))

lab <- subset(df, set = = ‘‘T’’, ‘‘lab’’,
drop = TRUE)
lab <- gsub(‘‘,| ’’, ‘‘’’, lab)
Yt <- as.factor(lab)

lab <- subset(df, set = = ‘‘V’’, ‘‘lab’’,
drop = TRUE)
lab <- gsub(‘‘,| ’’, ‘‘’’, lab)
Yv <- as.factor(lab)

library(caret)
fit <- train(Xt, Yt, method = ‘‘knn’’, tune-
Grid = data.frame(.k = c(3, 5, 7, 9)),
  trControl = trainControl(method = ‘‘cv’’, num-
    ber = 10, repeats = 10),
  preProcess = c(‘‘center’’, ‘‘scale’’, ‘‘pca’’))
```

The results of the training are stored in the object fit, and new data can be obtained by the predict method applied to this object. The final model with the best tuned parameters (stored in the finalModel slot of object fit) will be used for the prediction.

```
Yp<- predict(fit, newdata = Xv)
```

Table 4 shows the results of a benchmarking of the KNN algorithm. The fourth column reports the parameter k of the best tuned KNN model, and the last two columns contain the accuracy measure for the training and validation set respectively. The accuracy was computed as the ratio of gas classes correctly predicted by the model. We clearly observe that the model complexity, as expressed by greater values of k, increases with the greater scenario difficulty. It is reasonable that the discrimination of gas classes at higher levels of difficulty should require a more complex predictive model. The three models fitted to the scenarios at different difficulty levels also show differences in performance: the first model is able to classify 100% of the gas classes in both training and test sets, the second model shows quite good performance, and the third model performs poorly, giving the accuracy of 0.74 on the test set.

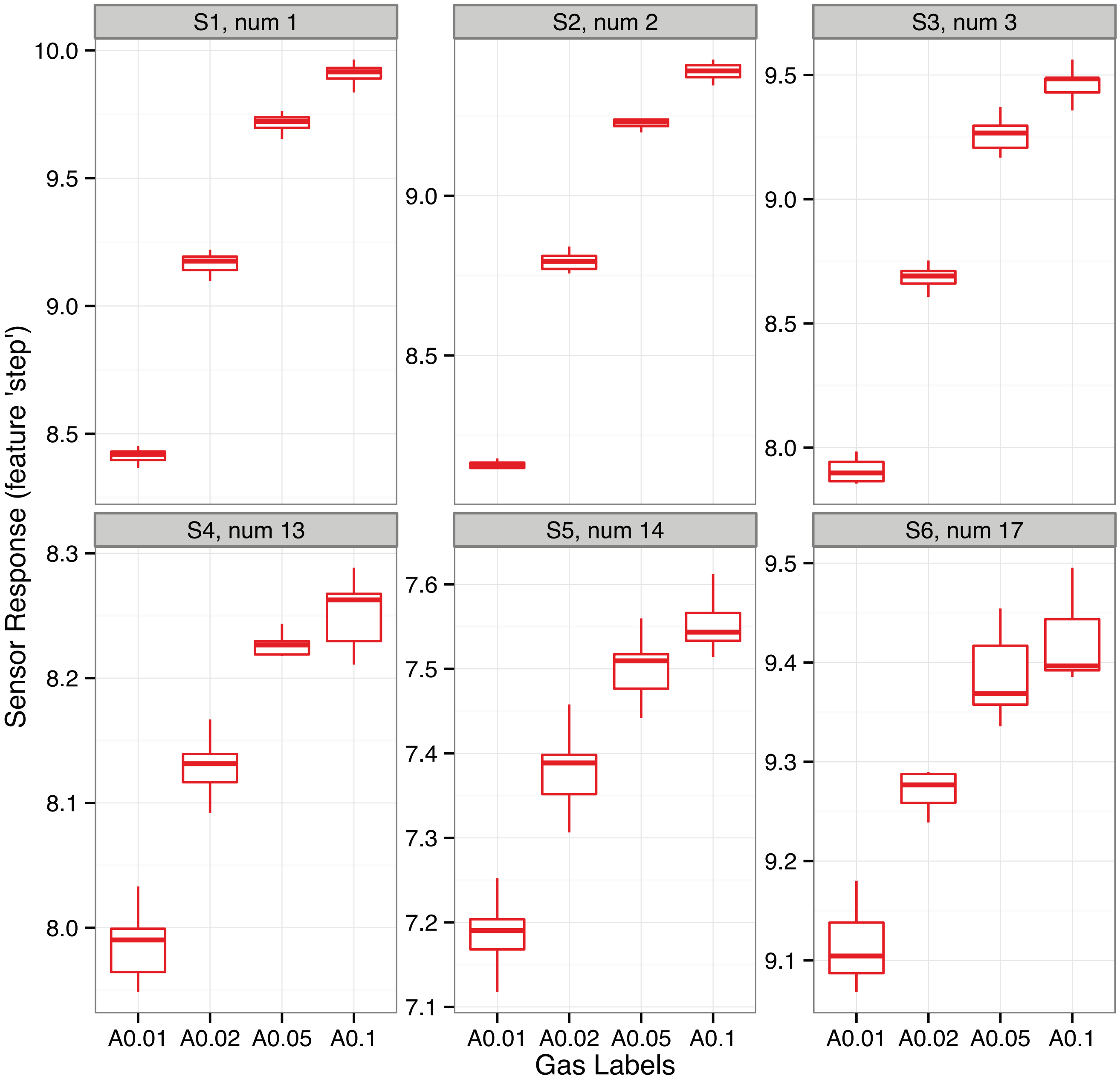

Figure 6. Boxplots for array of six sensors of types 1, 2, 3, 13, 14 and 17 show the distribution of sensor signals in response to analyte A at concentrations 0.01, 0.02, 0.05 and 0.1 vol.%. The concentration values were selected to cover the dynamic range of analyte A and to include the value in the saturation region. All the sensors show a non-linear response to analyte A at the selected concentration range. The three sensors of types 13, 14 and 17 show rather noisy responses. The plot is produced by the plotBoxplot method applied to the sensor array under drift-free conditions.
doi:10.1371/journal.pone.0088839.g006

Evaluation of Regression Algorithms

In this Section, we show an example of the regression scenario, which aims to quantify the concentration of a single analyte based on the sensor signals. To simulate data for benchmarking with the *chemosensors* package, one needs to define the analyte concentrations for the Scenario class and to configure a virtual sensor array for the SensorArray class. Further, one selects a method for the prediction model to perform the regression analysis on the simulated data, where the regression model will use the sensor signals as predictors and the concentrations as responses.

We consider two regression problems: one for analyte A at concentrations 0.01, 0.02, 0.05 and 0.1 vol.% and another for analyte C at concentrations 0.1, 0.4, 1 and 2 vol.%. The concentration range has been selected for each analyte in order to cover the dynamic range and to include the greatest concentration value in the saturation region. The following code shows the definition of a set of gas labels for each analyte.

```
conc.A <- c(0.01, 0.02, 0.05, 0.1)
set.A <- paste(''A'', conc.A)

conc.C <- c(0.1, 0.4, 1, 2)
```

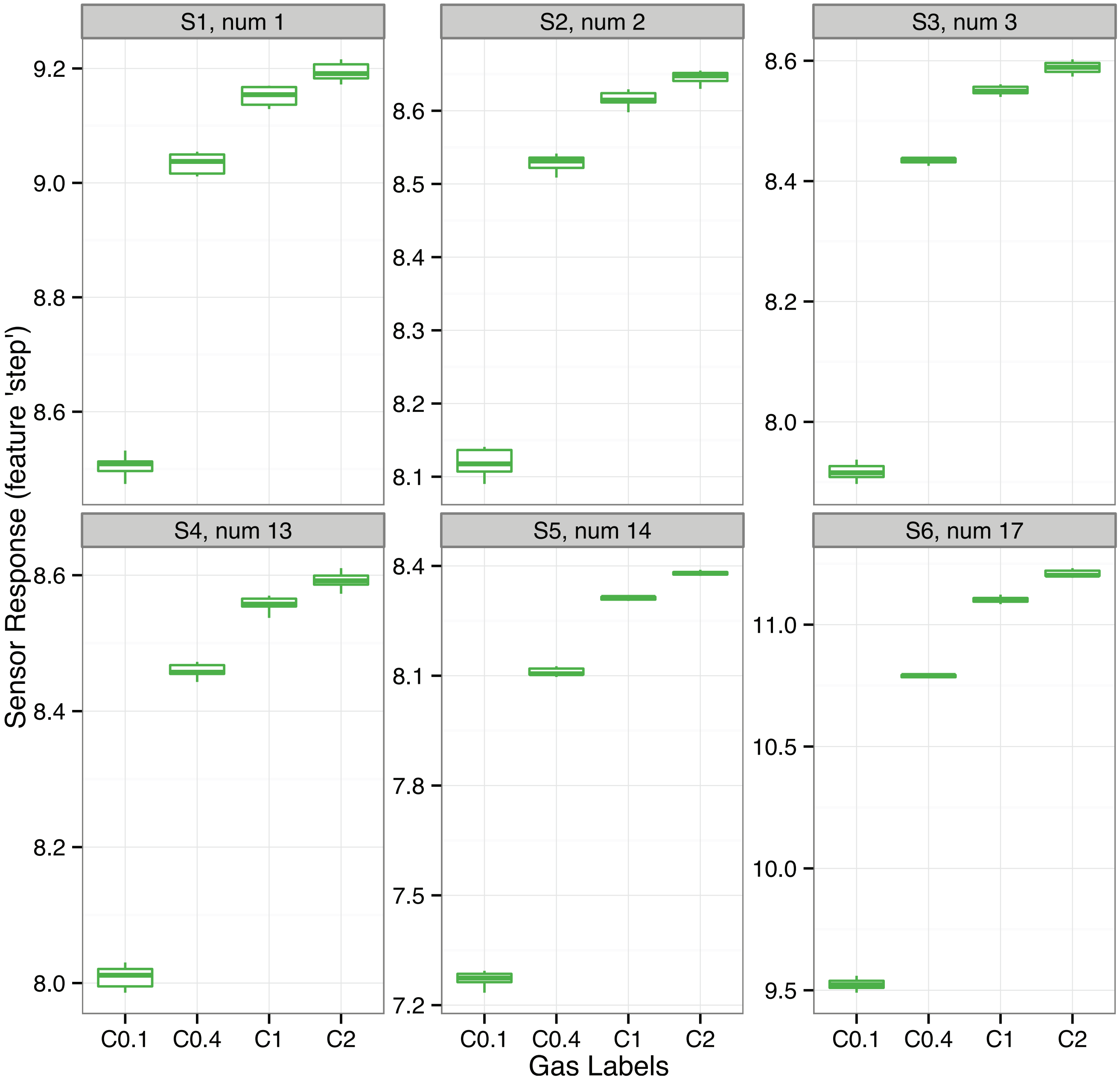

Figure 7. Boxplots for array of six sensors of types 1, 2, 3, 13, 14 and 17 show the distribution of sensor signals in response to analyte C at concentrations 0.1, 0.4, 1 and 2 vol.%. The concentration values were selected to cover the dynamic range of analyte C and to include the value in the saturation region. All the sensors show a non-linear response to analyte C at the selected concentration range. The plot is produced by the plotBoxplot method applied to the sensor array under drift-free conditions.
doi:10.1371/journal.pone.0088839.g007

```
set.C <- paste(''C'', conc.C)
```

We select the types of sensors by means of exploratory graphics available in the *chemosensors* package. We will also shorten the list of candidate types to six: 1, 2, 3, 13, 14 and 17, as they seem to be good candidates according to the characteristics of sorption affinity, as presented above. To evaluate these types of sensors in response to analytes A and C in different concentrations, we will create a virtual array composed of six sensors under drift-free conditions and apply the plotBox method, as shown in the code given below.

```
sa <- SensorArray(num=c(1:3, 13:14, 17),
dsd=0)

plotBox(sa, set=rep(set.A, 10), feature=''-
step'',
  sensors=1:6, sensor.names=''long'', gcol=-
TRUE, scales=''free_y'')
```

Figure 6 shows the box plots for the six types of sensors in response to four concentrations of analyte A. The same graphics for analyte C and its set of labels set.C is presented on Figure 7. All the sensors show a non-linear response to analytes A and C, as was expected due to the selection of the concentration ranges. In

Table 5. Performance on prediction of concentration of gas A under drift-free conditions.

Array	Types of sensors	Method	Parameters	RMSEP (train)	RMSEP (test)
1	1, 2, 3	pls	ncomp 9	0.0094	0.0208
1	1, 2, 3	svmRadial	C 2, sigma 10.7	0.0029	0.0039
2	13, 14, 17	pls	ncomp 2	0.0135	0.0133
2	13, 14, 17	svmRadial	C 2, sigma 91.2	0.0028	0.0105
3	1, 2, 3, 13, 14, 17	pls	ncomp 8	0.0086	0.0290
3	1, 2, 3, 13, 14, 17	svmRadial	C 2, sigma 20.1	0.0028	0.0045

Two methods, linear PLS and non-linear SVR, were tested on the regression task of analyte A given at concentration 0.01, 0.02, 0.05 and 0.1 vol.%. Three arrays composed of 24 sensors, different in the types of sensor, were compared in terms of the root-mean-square error in prediction (RMSEP). For each array, the non-linear models outperform the linear models. The first array and the SVR method yield the best performance.
doi:10.1371/journal.pone.0088839.t005

particular, the response to the lowest concentration is quite distinct from the others, whereas the responses to the two largest concentrations are quite close. One can also observe that the three sensors of types 13, 14 and 17 are very noisy in response to analyte A, this corresponds to sensor noise, as the drift noise has been suppressed in the sa array.

Since there is not an obvious choice of sensor type, we will try three different arrays composed of 24 sensor elements, as shown the following code.

```
sa1<- SensorArray(num=c(1:3), nsensors=24)
sa2<- SensorArray(num=c(13:14, 17), nsensors=24)
sa3<- SensorArray(num=c(1:3, 13:14, 17), nsensors=24)
```

In the first step, we simulate the data and store them in the df data frame, as shown in the following example of code given for the sa1 array and a set of gas labels set.A. We encode the Scenario object to make 100 repetitions of each gas label in both training and validation (test) set, this will allow us to have enough data to build a prediction model with validation by the 10-fold cross-validation procedure (10 repetitions).

```
sc <- Scenario(T=set.A, nT=100, V=set.A, nV=100, randomize=TRUE)
cf <- sdata.frame(sc)
conc <- getConc(sc)
sdata <- predict(sa1, conc, cores=2)
df <- sdata.frame(sa1, cf=cf, sdata=sdata)
```

In the second step, we train two regression models for each combination of sensor array and scenario. We will try one linear method based on Partial Least Squares (PLS) and another non-linear method based on Support Vector Regressor (SVR) with Gaussian radial basis function [13]. The following code shows the training of the two models fit1 and fit2, corresponding to the PLS and the SVR methods, respectively. The computation is given for the scenario for analyte A and the previously generated data stored df data frame.

```
Xt <- subset(df, set==''T'', select=snames(sa))
Xv <- subset(df, set==''V'', select=snames(sa))

Yt <- subset(df, set==''T'', select=''A'', drop=TRUE)
Yv <- subset(df, set==''V'', select=''A'', drop=TRUE)

library(caret)
fit1<- train(Xt, Yt, method=''pls'',
  tuneLength=24, preProc=c(''center'', ''scale''),
  trControl=trainControl(method=''cv'', number=10, repeats= 10,
  selectionFunction=''tolerance''))

fit2<- train(Xt, Yt, method=''svmRadial'',
  tuneLength=10, preProc=c(''center'', ''scale''),
```

Table 6. Performance on prediction of concentration of gas C under drift-free conditions.

Array	Types of sensors	Method	Parameters	RMSEP (train)	RMSEP (test)
1	1, 2, 3	pls	ncomp 2	0.3373	0.3384
1	1, 2, 3	svmRadial	C 0.5, sigma 237.7	0.0589	0.0837
2	13, 14, 17	pls	ncomp 7	0.2573	1.1317
2	13, 14, 17	svmRadial	C 0.5, sigma 74.9	0.0593	0.0790
3	1, 2, 3, 13, 14, 17	pls	ncomp 10	0.2365	2.8198
3	1, 2, 3, 13, 14, 17	svmRadial	C 0.5, sigma 114.5	0.0593	0.0877

Two methods, linear PLS and non-linear SVR, were tested on the regression task of analyte C given at concentration 0.1, 0.4, 1 and 2 vol.%. Three arrays composed of 24 sensors, different in the types of sensor, were compared in terms of the root-mean-square error in prediction (RMSEP). For each array, the non-linear models outperform the linear models. All three arrays show similar performance with the SVR method, and it is hard to pick the best array.
doi:10.1371/journal.pone.0088839.t006

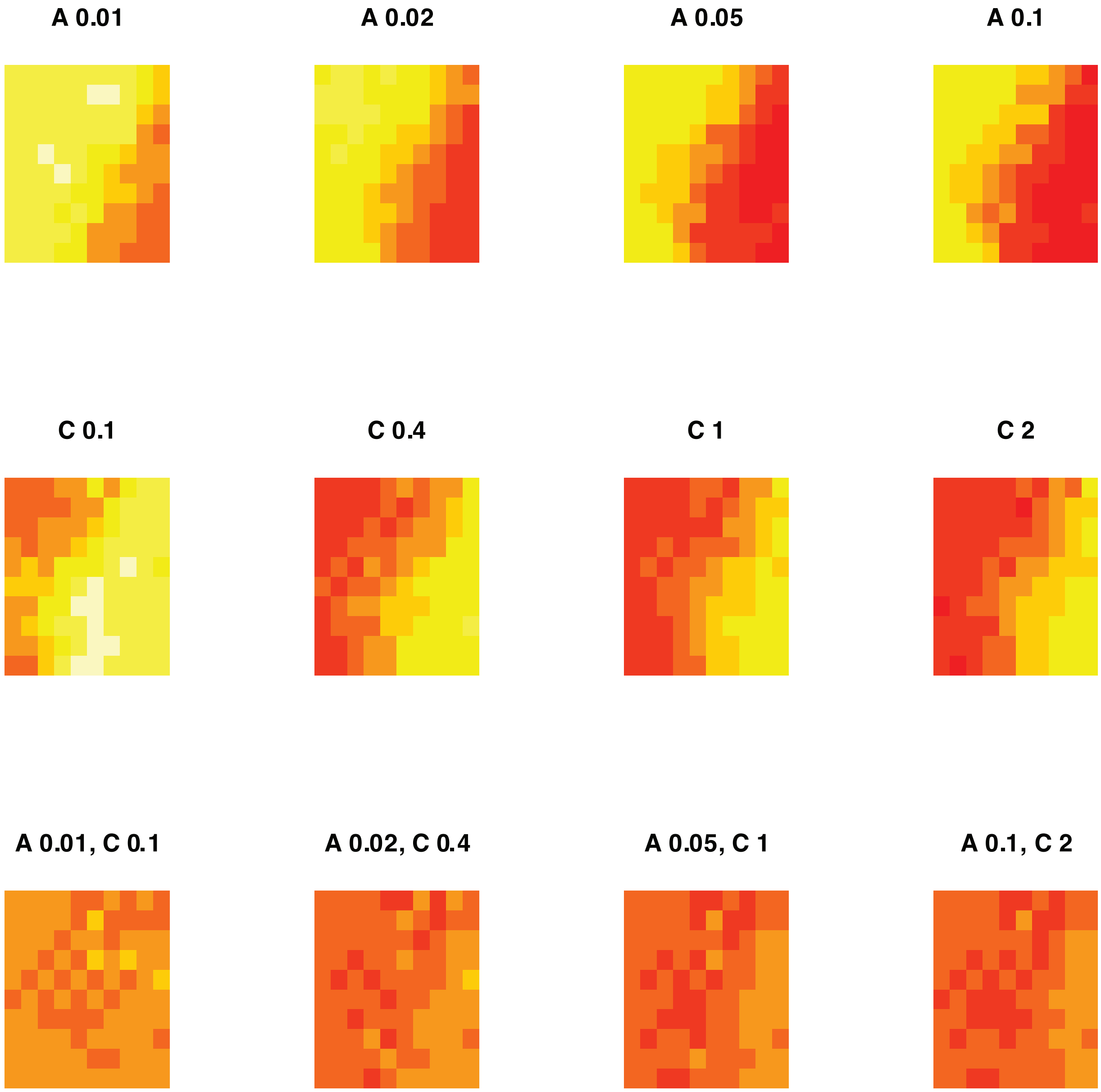

Figure 8. Heatmap of a self-organizing map (SOM) of size 7×7 showing the response to 12 different gases composed of analytes A and C. The map was constructed for the array of 1 K sensors based on the affinity coefficients computed per three analytes A, B and C for each sensor, as proposed in [23]. The response of sensor array for each gas was projected onto the map, and the colour on the heatmaps encode the magnitude of the signals in the SOM cells computed by averaging the signals from sensors assigned to the given cell. The activity of the SOM increases as the concentration of analytes increases (direction from left to right). The distribution of the SOM activity in response to different gases show that the right part of the map contain sensors with more affinity to analyte A, while the left part has sensor with more affinity to analyte C.
doi:10.1371/journal.pone.0088839.g008

```
trControl = trainControl(method = ''cv'', number = 10, repeats = 10,
selectionFunction = ''tolerance''))
```

To train both models, we pre-processed the sensor signals by performing centring and scaling operations and applied the 10-fold cross-validation procedure repeated 10 times. We also used the tolerance rule from the *caret* package to select the most appropriate model in the model tuning. This rule allows us to avoid overfitting of a regression model and suggests picking the simplest model which is within some percentage tolerance of the best model. The root-mean-square error in prediction (RMSEP) was used to evaluate the performance of the models and score them (the default error measure for regression analysis in the train function of the caret package). The fit1 model based on the PLS method has a single parameter ncomp which stands for the number of latent variables used in the regression. Tuning of the model was set to explore all the possible values for the ncomp parameter from 1 to 24. The fit2 model based on the SVR method has two parameters, the C parameter associated with the cost function and the parameter sigma of the kernel. By default, the train function of the *caret* package allows the estimation of the value of sigma from the data passed for training the model. Thus, tuning of the model was configured to explore 10 possible values of C parameter from 0.5 to 128, while the value of sigma parameter was pre-calculated and fixed in the procedure of model tuning.

For prediction of concentrations for new data, one applies the predict method to the model, as shown in the code below for the fit1 model and sensor signals stored for validation in Xv variable.

```
Yp<- predict(fit1, Xv)
```

The first results obtained for the initial experimental set up described above were confusing in terms of comparison among the arrays and the methods, and the error in both training and prediction was rather high and even comparable with the minimum concentration value of the analytes. The reason for experimental failure was explained by the substantial amount of drift-related noise observed in the sensor signals. Poor performance of the predictive models was attributable to the absence of any drift compensation procedure, this is a compulsory step in the most of the data processing methods in machine olfaction [4]. Hence, we repeated the step of data generation for all three sensor arrays sa1, sa2 and sa3 by setting the level of drift noise to zero. This strategy is reasonable, as the application of signal processing methods for drift compensation is outside the scope of this study, whose objective is the comparison of different arrays and regression methods on the quantification task for analyte concentrations.

Tables 5 and 6 summarize the results obtained from the drift-free experimental set up for analytes A and C, respectively. Three arrays sa1, sa2 and sa3 are numbered by indexes 1, 2 and 3, as given in the first column. All the arrays are composed of 24 sensors and differ in the types of sensors, which are listed in the second column. The regression method and the best set of parameters for it (as derived after the model tuning) are given in the next two columns. The last two columns report the RMSEP for the training and test sets.

The comparison between PLS and SVR methods in terms of RMSEP values clearly shows that the non-linear models outperform the linear models for each of the arrays. The difference is more noticeable for analyte C than for analyte A. That seems reasonable, as Figures 6 and 7 show that sensor signals in response to analyte C exhibit more a non-linear structure than in response to analyte A (at the given concentrations of the analytes). The best performance (in terms of RMSEP for the test set) for the task of quantification of analyte A is exhibited by the sa1 array and the SVR model. The sa2 array, composed of sensors from different types than sa1, shows a significantly higher error in prediction; this is assumed to be related to a higher level of the sensor noise in response to analyte A, as was depicted on Figure 6. The performances (in terms of RMSEP for the test set) of the three arrays, for the task of quantification of analyte C, are very similar for the SVR model, and it is difficult to select a preferred configuration of array for this task.

Example of a Large-scale Simulation

In this Section, we show an application of the *chemosensors* package in performing biologically-inspired data processing of sensor array data. In particular, we will be interested in the modelling of chemotopic convergence of receptor neurons occurring in the early olfactory pathway. We will implement a simple neuromorphic model based on the Self-Organizing Map (SOM) technique and will repeat the experiment conducted in [23] by using data produced from a virtual sensor array.

Since neuromorphic models require a large number of sensors in the array and a sufficient level of diversity across the sensors, we will create an array constructed of 1 K elements parametrized with all 17 sensor types and a beta parameter of diversity set to 5 (the default value of beta is 2).

```
sa <- SensorArray(num=1:17, nsensors=1000,
beta=5)
```

Then we compute the matrix of affinity characteristics aff for each sensor and for each analyte by the method given in [23]. Further, the aff matrix will be used to evaluate the SOM of size 10×10 by means of the kohonen package, as given in the code below.

```
aff <- computeAffinity(sa, method=''in-
verse'', norm=''norm'')

library(kohonen)
map<- som(scale(aff),
  grid=somgrid(xdim=10, ydim=10, topo=''r-
    ectangular''), rlen=500)
```

In the next step, we use three types of gas labels: pure analyte A at concentration of 0.01, 0.02, 0.05 and 0.1 vol.%, pure analyte C at concentration of 0.1, 0.05, 1 and 2 vol.%, and four binary mixtures of analytes A and C. We will suppress all the noise models by means of the nsd method and will run the simulation of sensor signals on a machine with 8 cores to get results in a reasonable amount of time.

```
set.A <- paste(''A'', c(0.01, 0.02, 0.05, 0.1))
set.C <- paste(''C'', c(0.1, 0.4, 1, 2))
set.AC <- paste(set.A, set.C, sep='', '')
set <- c(set.A, set.C, set.AC)

sc <- Scenario(set)
conc <- getConc(sc)

nsd(sa) <- 0

sdata <- predict(sa, conc, cores=8)
df <- sdata.frame(sa, conc=conc, sdata=s-
data, feature=''step'')
```

The generated sensor array data are stored in the df data frame with 12 rows, this corresponds to 12 gas labels stored in the set variable. Further, we project signals from 1 K sensors onto the 100 cells of the SOM. Figure 8 show the heatmaps of the SOM, where the colours encode the magnitude of the sensor signals in the SOM cells computed by averaging the signals assigned to the given cell. We observe an increasing activity of the map, as expressed in the change from yellow to red, as the concentration of analytes increases in the gas (direction from left to right). Another observation is related to the distribution of sensors or sensor types across the map. The right part of the map is more active in response to analyte A, and the left part of the map shows more activity in response to analyte C. The heatmaps presented in the lowest raw of the figure correspond to the measurements of the binary mixtures, and the SOM maps show activity of both left and right parts of the map.

Conclusions

The *chemosensors* package is a new R package for data simulation targeted at generating gas sensor array data for signal and data processing in machine olfaction applications. The package contains a set of simulation models organized as S4 classes, which are unified in the main class SensorArray. This class allows the creation of a virtual sensor array, serves as a data generation tool, and offers a large list of configuration parameters. The class Scenario makes it easier to define scenarios and then generate data together with the virtual array. In summary, the *chemosensors* package provides a compact and extensively configurable work-

flow for data generation, supports parallelization of large-scale computations and offers many graphical facilities to explore sensor array data. In future, the proposed computational framework for the simulation of sensor arrays can be extended to new reference data sets of different types of sensors and/or of different combinations of analytes, that, in turn, will allow addressing new challenges in machine olfaction, for instance, simulation of the sensor response for high-dimensional multicomponent chemical input.

Supporting Information

File S1 Ten scenarios for machine olfaction in the framework of the NEUROChem project [3].
(PDF)

Appendix S1 Parameterization of Simulation Models.
(PDF)

Appendix S2 Quantitative Comparison with Chemical Sensor Data.
(PDF)

Acknowledgments

The authors would like to acknowledge the effort made by the anonymous reviewers of this manuscript which have greatly contributed to the improvement of the quality of the work. The authors also wish to thank Dr Oleg N. Osychenko and Mrs Marta Oliete for their assistance in revising the English of this manuscript.

Author Contributions

Conceived and designed the experiments: AZ APL. Performed the experiments: AZ APL. Analyzed the data: AZ APL. Contributed reagents/materials/analysis tools: AZ APL. Wrote the paper: AZ APL.

References

1. Lander ES, Linton LM, Birren B, Nusbaum C, Zody MC, et al. (2001) Initial sequencing and analysis of the human genome. Nature 409: 860–921.
2. Persaud K, Dodd G (1982) Analysis of discrimination mechanisms in the mammalian olfactory system using a model nose. Nature 299: 352–355.
3. Marco S, Gutiérrez-Gálvez A (2012) Signal and Data Processing for Machine Olfaction and Chemical Sensing : A Review. IEEE Sensors Journal 12: 3189–3214.
4. Pearce T, Schiffman S, Nagle H, Gardner J (2003) Handbook of Machine Olfaction - Electronic Nose Technology. John Wiley & Sons.
5. Gutiérrez-Osuna R (2002) Pattern analysis for machine olfaction: a review. IEEE Sensors Journal 2: 189–202.
6. Di Natale C, Martinelli E, Paolesse R, D'Amico A, Filippini D, et al. (2008) An Experimental Biomimetic Platform for Artificial Olfaction. PLoS ONE 3: e3139.
7. Berna AZ, Anderson AR, Trowell SC (2009) Bio-Benchmarking of Electronic Nose Sensors. PLoS ONE 4: e6406.
8. Vergara A, Vembu S, Ayhan T, Ryan Ma, Homer ML, et al. (2012) Chemical gas sensor drift compensation using classifier ensembles. Sensors and Actuators B: Chemical : 1–10.
9. Raman B (2005) Sensor-based machine olfaction with neuromorphic models of the olfactory system. Ph.D. thesis, Texas A&M University.
10. Fonollosa J, Gutierrez-Galvez A, Lansner A, Martinez D, Rospars J, et al. (2011) Biologically Inspired Computation for Chemical Sensing. Procedia Computer Science 7: 226–227.
11. Ziyatdinov A, Fernández Diaz E, Chaudry A, Marco S, Persaud K, et al. (2013) A software tool for large-scale synthetic experiments based on polymeric sensor arrays. Sensors and Actuators B: Chemical 177: 596–604.
12. R Core Team (2013) R: A Language and Environment for Statistical Computing. Available: http://www.r-project.org/.
13. Zeileis A (1999) Collaborative Software Development Using R-Forge : 9–14.
14. Eriksen MMA, Svante P (2012) fwsim: Fisher-Wright Population Simulation. Available: http://cran.r-project.org/package = fwsim.
15. Vigeland MD (2012). IBDsim: Simulation of chromosomal regions shared by family members. Available: http://cran.r-project.org/package = IBDsim.
16. Montana G (2012) hapsim: Haplotype Data Simulation. Available: http://cran.r-project.org/package = hapsim.
17. Alfons A, Templ M, Filzmoser P (2010) An Object-Oriented Framework for Statistical Simulation: The R Package simFrame 37.
18. Ziyatdinov A, Marco S, Chaudry A, Persaud K, Caminal P, et al. (2010) Drift compensation of gas sensor array data by common principal component analysis. Sensors and Actuators B: Chemical 146: 460–465.
19. Ziyatdinov A (2012) Home page of chemosensors package. Available: http://chemosensors.r-forge.r-project.org/.
20. Kuhn M (2008) caret Package 28.
21. Wehrens R, Buydens LMC (2007) Self- and Super-organizing Maps in R : The kohonen. Journal of Statistical Software 21.
22. Hastie T, Tibshirani JFR (2009) The Elements of Statistical Learning: Data Mining, Inference, and Prediction. Springer, second edition. Available: http://www-stat.stanford.edu/tibs/ElemStatLearn/.
23. Raman B, Gutiérrez-Gálvez A, Perera-Lluna A, Gutiérrez-Osuna R (2004) Sensor-based machine olfaction with a neurodynamics model of the olfactory bulb. 2004 IEEE/RSJ International Conference on Intelligent Robots and Systems (IROS) (IEEE Cat No04CH37566) : 319–324.
